# Novel Sewer Defect Prediction Leveraging Advanced Machine Learning (ML) Models

**DOI:** 10.1002/wer.70338

**Published:** 2026-03-19

**Authors:** Vannary Seng, Barbara J. Lence, Sudhir Kshirsagar, Srujana Rangapuram, Pavan Saranguhewa

**Affiliations:** ^1^ Department of Civil Engineering University of British Columbia Vancouver British Columbia Canada; ^2^ Global Quality Corp. (GQC) Covington Kentucky USA; ^3^ Infosys Ltd. Richardson Texas USA; ^4^ Electrical Engineer II, Excelitas Technologies Gaithersburg Maryland USA

## Abstract

A novel approach to sewer network assessment is presented that uses artificial intelligence (AI)/machine learning (ML) to predict infiltration and structural defect occurrences in each pipe instead of estimating the traditional criteria‐based overall pipe condition or likelihood of failure. A comparative analysis of four decision tree‐based ML models, and their use in predicting the defect locations in sewer networks, is presented. The models are developed using data from closed‐circuit television (CCTV) inspections coupled with additional pipe information and inspection reports. The ML approach uses such information from two utilities to create utility‐specific defect prediction models. The class imbalance in the data, due to more defects than nondefects, is addressed with three methods, and the hyperparameters, settings that define the model architecture, are optimized via a repeated stratified k‐fold cross‐validation grid search. The performance of the models is assessed using the area under the receiver operating characteristics (AUC‐ROC) and precision recall (AUC‐PR) curves. LightGBM‐based models, with the cost‐sensitive learning method for addressing class imbalance, show the best performance overall when predicting either types of defects for both utilities. The best performing model achieves an AUC‐ROC of 0.79 and an AUC‐PR of 0.62. For the two utilities investigated, an application of SHapley Additive exPlanations (SHAP) shows that the most important features for indicating both types of defects are “pipe location” and “pipe age.”

## Summary

- Machine learning models are developed to predict infiltration and structural defect occurrences in sewers based on pipe characteristics and locations.
- Models predict specific defects rather than overall pipe condition, making them suitable for data from various condition assessment standards.
- SHapley Additive exPlanations (SHAP) analyses are applied to identify pipe characteristics that are most associated with the occurrence of infiltration and structural defects.
- Models may be used to undertake the asset management of sewer networks.

## Graphical abstract

An evaluation of four decision tree‐based machine learning models is conducted to predict infiltration and structural defects occurrences in sewer networks using pipe characteristics and locations. Utility‐specific models are developed for two Western Canada utilities showing pipe location and age, two of the key predictors of both defect types.

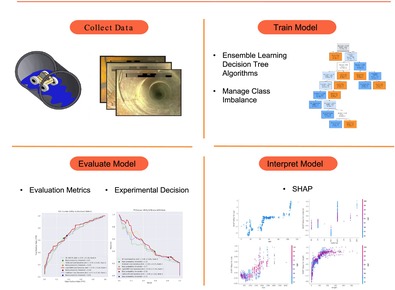

## 1 Introduction

The majority of sewer networks in North America date to the 1960s and are facing increased loadings related to population growth and climate change, exacerbating combined sewer overflows, operational problems, and their commensurate environmental and societal consequences. Asset management strategies for such aging networks are hindered by the significant capital investment required and the inherent difficulty in assessing the condition, and thus service life, of buried sewers (Harvey and McBean 2014b; Tscheikner‐Gratl et al. 2020). Typically, sewer condition is assessed by filming the sewer interior with a remotely operated closed‐circuit television (CCTV) rover, inspecting the CCTV videos, and estimating the condition grade based on the quantity and types of defects observed and their intensity. However, the sewer inspection process using CCTV rovers is labor intensive, time consuming, and not feasible for all types of sewer pipes, such as small diameter pipes, pipes without ready access points, or pipes with sharp bends. In this paper, we use information from CCTV inspection reports and other pipe data information to create data‐driven binary classification machine learning (ML)‐based models for predicting the likely presence of infiltration and structural defects which are crucial for maintaining structural integrity and pipe hydraulic capacity, thus circumventing the shortfalls of traditional sewer condition assessment procedures.

Researchers have developed sewer deterioration models to predict the condition of uninspected sewers. These models are based on relationships between deterioration factors, that is, physical, environmental, and operational variables, and estimated condition grades for inspected pipes in the network, where such relationships may be mechanistic, statistical, or based on ML algorithms (Kley and Caradot 2013). With growing interest in the application of ML, researchers have developed ML‐based sewer deterioration models to predict the condition grades or likelihood of failure of sewers based on data in CCTV reports, field observations, and condition assessment standard inspection standards, such as the National Association of Sewer Service Company (NASSCO) Pipeline Assessment and Certification Program (PACP) and the Water Research Center Manual of Sewer Condition Classification (WRc MSCC).

Najafi and Kulandaivel (2005) explore the application of an artificial neural network (ANN)‐based algorithm to capture the relationships between sewer length, size, material, age, depth of cover, slope, type of sewer, and pipe condition ranking, where the latter ranges from 1 (*best*) to 5 (*worst*). The importance of an individual variable, also referred to as a feature, is found by excluding the interested variable from a given model development and evaluating the model performance. All such models are then compared, and the importance rankings of the variables correspond to the relative performance of the respective models. Tran et al. (2006) apply a probabilistic neural network, which performs better than a discriminant model, though is difficult to interpret. Khan et al. (2010) investigate the importance of sewer features for indicating sewer condition by evaluating the sensitivity of the sewer condition predicted to a range of values for the sewer features. Mashford et al. (2011) use additional features, such as soil corrosiveness and soil/groundwater characteristics to develop a support vector machine (SVM)‐based model. This study finds that, given the limited data, the additional variables do not improve model performance.

Sousa et al. (2014) show that an ANN algorithm outperforms an SVM for predicting sewer condition and highlight the importance of considering uncertainty in model performance when selecting the best model. Harvey and McBean (2014a) show that a decision tree algorithm outperforms an SVM and reduces the five structural condition ranks to two ranks: good (Ranks 1–3) and bad (Ranks 4–5). Random forest, RF (Breiman 2001), which employs an ensemble of decision trees, has become the most popular algorithm for providing high performance in predicting sewer condition (Caradot et al. 2018; Harvey and McBean 2014b; Hernández et al. 2018; Laakso et al. 2018; Rokstad and Ugarelli 2015; Vitorino et al. 2014). Syachrani et al. (2013) develop a model to predict pipe real‐age using reported structural condition and sewer characteristics. The study compares regression, ANN, and decision tree‐based models and finds that the latter model outperforms the others and offers a visual representation of the decision‐making process, allowing for easier interpretation of the results.

While sewer deterioration models are useful in predicting the sewer condition, condition grades provided in CCTV inspection reports are subjective and dependent on inspector interpretation and experience and thus are subject to uncertainty (Rahman and Vanier 2004; Rokstad and Ugarelli 2015; Salihu et al. 2023). In addition, estimated condition grades only provide limited information due to the aggregation process. For example, two pipes with the same condition grade may have experienced different failure modes, and these can often be explained by different deterioration factors (Rokstad and Ugarelli 2015). Malek Mohammadi et al. (2020) review the findings of work from 2001 to 2019, identify several variables that affect condition, and note cases in which past studies obtain contradictory results.

Most recent research focuses on predicting pipe failures where input data are field observations rather than from CCTV inspection reports. Tavakoli et al. (2020) develop a RF‐based model to predict whether the pipe is due for inspection or not, based on 160,370 observation data points. The overall accuracy is high, but the model has problems with imbalanced data, where pipes with good condition are in the majority. Hoseingholi and Moeini (2023) conduct a comparative study between ANN‐based and genetic programming (GP)‐based regression models to predict the failure rate per length by dividing data into three different datasets: all training data, split by time, and split by zone. The study finds that the GP‐based regression models perform better than the ANN‐based models. Kizilöz (2024) conducts a comparative study of four algorithm‐based classifier models including ANN, RF, gradient boosting machine, and ANN‐RF hybrid models, along with various feature selection algorithms, to predict pipe failure. The study results are compared with the two aforementioned studies as well as five others that use various prediction algorithms. Although the model accuracy is high, the study suffers from data thinness. These failure models, as well as many of the condition assessment models, suffer from limited and imbalanced data, that is, where the proportion of the condition grades of the pipes in the data set are not uniform. Caradot et al. (2018) note that such imbalances lead to biased predictions.

In this study, rather than predicting pipe condition or failure rates, ML‐based binary classification models that predict specific defects at the individual pipe level are developed to target major maintenance concerns facing utilities, specifically infiltration and structural defects. The structural condition of a pipe is associated with its ability to resist external loads which could lead to deformation and collapse (Malek Mohammadi et al. 2020), and these, along with infiltration, if left unchecked, will reduce the hydraulic capacity of sewers and exacerbate further structural problems (Najafi and Kulandaivel 2005). Prediction of specific defects provides relevant information to utilities as they plan their asset improvement strategies, and the ML‐based models identify complex relationships between data from diverse sources and defect predictions. We also explore methods for accounting for class imbalance, the analysis of these models with common graphical techniques that distinguish them considering their performance uncertainty, and the analysis of the most important features for indicating the defects.

Models based on the following four well‐known ensemble learning decision tree algorithms are explored: RF, eXtreme gradient boosting, XGBoost (Chen and Guestrin 2016), light gradient boosting machine, LightGBM (Ke et al. 2017), and category boosting (CatBoost) (Prokhorenkova et al. 2019). Three methods for managing imbalanced data are examined: the cost‐sensitive learning, an oversampling, and an undersampling method. Two utility‐specific models, one for infiltration and one for structural defects, are developed and evaluated for sewer inspection data for two utilities in Western Canada with widely varying pipe characteristics.

The models are compared in terms of the resulting area under the receiver operating characteristics (AUC‐ROC) and precision recall (AUC‐PR) curves. The Mann–Whitney *U* test, which considers the uncertainty of each model performance, is used to determine whether the models are distinctly different. SHapley Additive exPlanations (SHAP) (Lundberg and Lee 2017) analyses are applied to determine the features that are important in predicting the presence of the infiltration and structural defects. This analysis provides a visual and easily interpreted representation of feature importance, and the exploration of the related partial dependence plots provides insights into the impact of various model predictor variables on different defects.

## 2 Methodology

For a sewer pipe with a given pipe identification number (pipe ID), the ML‐based binary classification models developed herein predict whether infiltration or structural defects are expected to occur based on the pipe characteristics as obtained from the utility geographic information system (GIS) databases and the CCTV inspection reports. Inputs to the models include pipe midpoint location, age, size, length, shape, and material, as model predictor variables or features. The model outputs, or model target variables, are whether or not a known defect of a given type is located in the pipe; for example, for the infiltration binary classification model, the output classes are infiltration expected and no infiltration expected.

### 2.1 Model Predictor Variables

CCTV inspection reports and sample pipe information from two utilities, referred to as Utilities A and B, are summarized in Table 1. Utility A contains data based on both PACP and WRc MSCC, and Utility B contains data based on PACP only. Column 1 provides the name of the characteristic, and Columns 2–4 provide values for the dataset specified. The reports cover a portion of the entire network, where the network of Utility A consists of 400 km of sanitary sewage pipes and that of Utility B of 553 km of combined and sanitary sewage pipes (*note:* the data provided by Utility B do not distinguish between sewer types).

**TABLE 1:** CCTV and sample pipe information.

Utility	Utility A	Utility A	Utility B
Condition assessment standard	PACP	WRc MSCC	PACP
No. CCTV inspection reports	799	2839	1376
Inspection years	2018–2022	1989–2015	2017–2022
No. pipes in sample (total length)	799 (48 km)	2839 (172 km)	1837 (184 km)
No. unique pipe ID^a^ (total length, % of network total length)	698 (42 km, 10%)	2231 (131 km, 33%)	1439 (137 km, 25%)
Age range (years)	1–71^b^	1–71^b^	1–122
Types of pipe material^c^ (% of pipes of this type in sample)	AC (2%),	AC (17%),	AC (3%),
	CP (12%),	CP (13%),	CP (58%),
	PVC (1%),	PE (< 1%),	PE (< 1%),
	RCP (< 1%),	PVC (5%),	PVC (11%),
	SP (< 1%),	VCP (26%),	RCP (25%),
	VCP (4%),	XXX (< 1%),	SP (< 1%),
	ZZZ (< 1%)	ZZZ (< 1%)	VCP (< 1%),
			ZZZ (2%)
Types of pipe shape^c^	C	C	A, B, C, E, H, R
Pipe height/diameter (mm)	150–600	150–700	200–4050

Abbreviations: A, arched; AC, asbestos cement; B, barrel; C, circular; CP, concrete pipe; E, egg‐shaped; H, horseshoe; no., number; PE, polyethylene; PVC, polyvinyl chloride; R, rectangular; RCP, reinforced concrete pipe; SP, steel pipe; VCP, vitrified clay pipe; XXX, not known; ZZZ, other.

^a^ Due to some pipes being inspected more than once.

^b^ Estimated by utility.

^c^ Based on PACP condition assessment standard (NASSCO 2022).

The data in common for both utilities are selected as the predictor variables, and these are listed in Table 2. Whether the data type is numerical or categorical, the data description and the range and statistical values of the data are provided. To identify the pipe location, age, and length and whether or not a defect of a given type is present in the pipe, the location and type of all defects are required. CCTV inspection often takes place between adjacent manholes (NASSCO 2022; WRc 2001) and may include inspection of several pipes, with different pipe IDs, in series. Inspection reports provide the set of pipe IDs of the pipes viewed in sequence, the location of any defect from the starting point of the inspection, and the length of the inspection. The location of the defect and its corresponding pipe ID are determined by noting the distance to defect relative to the starting point in the inspection report and tracing the pipe IDs sequentially along pipe polylines according to their order in the GIS database. Should only the ending point of the inspection be available in the GIS database, the defect may be located relative to the ending point by tracing them sequentially along the polyline in the reverse order of the pipe IDs. Like most utilities in the reviewed literature, Utilities A and B do not have extensive records regarding environmental information (Malek Mohammadi et al. 2020). In lieu of such information, the location of the pipe centroid, represented as two variables, is chosen as it may help relate the model findings to nearby features such as soil types, road cover, and traffic loads.

**TABLE 2:** Model predictor variables.

Variable name (unit)	Data type	Description	Min/max/mean/median/std.d.
*X*‐coordinate (m)	Numerical	*x*‐coordinate of pipe's centroid	Undisclosable information
*Y*‐coordinate (m)	Numerical	*y*‐coordinate of pipe's centroid	Undisclosable information
Age (year)	Numerical	Pipe age at the time of inspection	A:1 B:0/
			A:71 B:122/
			A:45 B:53/
			A:46 B:51/
			A:13 B:23
Pipe size (mm)	Numerical	Pipe height/diameter	A:150 B:200/
			A:700 B:4050/
			A:190 B:1027/
			A:150 B:900/
Pipe length (m)	Numerical	Pipe length	A:71 B:666
			A:1.57 B:0.11/
			A:136 B:566/
			A:60 B:100/
			A:59 B:90/
			A:27 B:80
Pipe shape	Categorical	Type of pipe shape	Category distribution in Table 1
Pipe material	Categorical	Type of pipe material	Category distribution in Table 1

Owing to repairs that may have taken place over time, a number of cases occur in which the pipe material, size, or shape documented in the CCTV inspection reports are different from the material associated with the respective pipes as reported in the GIS database. Since the information in the CCTV inspection reports is more current than the GIS data and is considered more reliable, the source of size, shape, and material information is the inspection reports. For Utility A, in some cases, pipe sizes (42 pipe samples) and pipe materials (17 pipe samples) are not provided in the inspection reports, and in these cases, this information is obtained from the GIS database for the given pipe ID.

### 2.2 Model Target Variables

While the PACP and WRc MSCC condition assessment standards are different, they are both comprised of a defect coding system which describes the defect type, the severity of defects, and the defect direction or location on the circumference of the pipe (NASSCO 2022; WRc 2001). While a variety of defect types are typically noted, for purposes of the ML models developed herein, the relevant defects are sorted into two groups; that is, (i) all defects associated with infiltration are considered one group, regardless of the level of distress, direction, and circumferential location identified; and (ii) structural defects that may lead to collapse, including cracks, fractures, breaks, holes, deformations, and collapses, are the other. While some information regarding defect severity is not considered, this approach is undertaken in order to align the defect codes of the two condition assessment standards, to reduce data thinness, and to account for the uncertainty resulting from the subjectivity of inspectors' interpretations.

The target variables in the binary classification ML‐based models are whether or not either infiltration or structural defects are observed in the inspection. Different ML‐based models are developed that relate the values of model predictor variables to one or the other of these model target variables; thus, there are two sets of binary classification ML‐based models for each utility. The model sets are comprised of models that are based on one of four learning algorithms, one of three methods for addressing imbalanced data, and one of the two target variables, that is, whether or not the given defect exists.

### 2.3 Model Development

The model predictor and target variable values for each of the pipes viewed in the inspection reports (799 + 2839 = 3638 and 1376, for Utilities A and B, respectively) were used to develop the ML‐based models. For each model, 80% of all data samples, that is, training and validation data, are used to train and tune the given algorithm, in order to determine the relationships between model predictor and target variables and to calibrate the model settings, and 20% of all data samples, that is, testing data, are used to evaluate the overall model performance after the training process is complete and to support model comparisons. While the data are not divided by years of record, the data are stratified split to preserve the distributions of the target classes in both the training and the testing datasets as shown in Table 3, where I and SD represent infiltration and structural defects, respectively.

**TABLE 3:** Number of training and testing data.

Classification model	No. of training data (target class to no target class ratio)	No. of testing data (target class to no target class ratio)
Utility A‐I	2910 (433:2477)	728 (108:620)
Utility A‐SD	2910 (860:2050)	728 (215:513)
Utility B‐I	1469 (430:1039)	368 (108:260)
Utility B‐SD	1469 (297:1172)	368 (74:294)

Abbreviations: I, infiltration; SD, structural defects.

#### 2.3.1 Ensemble Learning Decision Tree Algorithms

The architecture of all four decision tree algorithms consists of a collection of decision trees and employs an ensemble method to combine results from this collection to determine the final model prediction. The RF algorithm uses a bagging method in which multiple decision trees are built in parallel based on a random subset of the data, which is obtained by sampling with replacement from the training data, and the predictions of these trees are aggregated to provide the trained RF‐based model. The XGBoost, LightGBM, and CatBoost algorithms use a gradient boosting method in which multiple decision trees are built in sequence and improve upon each other, all using the entire dataset. The other differences among these algorithms relate to their method for guiding the tree branching process, that is, how the data are split and evaluated conditioned on the values of the model predictor variables, their computational efficiencies, the degree to which they “overfit the data’, and their approach for supporting categorical variables.

A key factor that contributes to the efficiency of RF is its branching process which is guided by evaluating the information gain, that is, prediction improvement, for any subset of model predictor variables and their values, measured with the Gini index or entropy. Its bagging ensemble method is known to be more robust than boosting against overfitting and noisy data (Fawagreh et al. 2014). It also requires a smaller number of hyperparameters to be tuned than the other algorithms.

XGBoost achieves increased computational efficiency by presorting the data in the order of the model predictor variable values, before undergoing the tree branching process (Chen and Guestrin 2016). Thereafter, each possible condition split is pursued, and the information gain of predictions from each branch is based on the gradient and Hessian of the loss function; for example, for binary classification models, a binary log loss function is used. Compared with RF, XGBoost is more susceptible to overfitting if the hyperparameters are not properly turned.

LightGBM realizes even more computational efficiency by applying gradient‐based one‐side sampling (GOSS) and exclusive feature bundling, EFB (Ke et al. 2017). To reduce the training dataset, GOSS retains all data points with large loss function gradients and randomly selects and retains a sample of those with small loss function gradients. To reduce the model predictor variable set, EFB evaluates whether any of the variables are mutually exclusive, that is, a variable is mutually exclusive from other variables, if when the variable is nonzero, the other variables have a value of zero.

Three of the four algorithms examined use level‐wise growth (Korf 1985), where all immediately subsequent nodes (so‐called leaf nodes) at a given branch depth are split and evaluated before proceeding to the next branch depth. Unlike these, LigthGBM uses leaf‐wise growth (Friedman et al. 2000), where the leaf node corresponding to the highest improvement is pursued first, resulting in smaller and faster models than the other three algorithms. However, this may lead to overfitting when the dataset is small if the maximum depth of a tree is not sufficiently tuned.

Unlike the other three algorithms that are asymmetric in structure, CatBoost builds symmetric decision trees in which the predictor variable and variable value‐pair resulting in the smallest loss function is applied to all the leaf nodes at a given depth. This helps control overfitting and reduce prediction time. In addition, CatBoost further reduces its potential for overfitting by using ordered boosting. Unlike the classic boosting method applied in XGBoost and LightGBM that uses the same data points to train the model and calculate the model fitting residuals, ordered boosting uses one subset of data to train and a different subset to calculate the residuals (Prokhorenkova et al. 2019).

For all ML‐based models, two model predictor variables are categorical, namely, pipe material and shape, as listed in Table 2. At the time of the study, in the version of RF and XGBoost algorithms used, a preprocessing step to convert categorical variables to numerical variables is required. One common process of conversion, known as one‐hot encoding, involves creating a variable for each possible category of the variable, where a binary assignment is used, that is, 1 or 0, indicating whether or not that category describes the categorical variable. In contrast, both LightGBM and CatBoost can support categorical variables internally without data preprocessing.

The *sklearn.ensemble. RandomForestClassifier* API in Python and the *xgboost*, *lightgbm*, and *catboost* in Python packages are used to implement RF, XGBoost, LightGBM, and CatBoost algorithms, respectively. To address categorical variables, one‐hot encoding is implemented for the RF‐ or XGBoost‐based models.

#### 2.3.2 Methods for Addressing Imbalanced Data

For binary classification ML‐based models, unless otherwise addressed, nonuniformly distributed target variable values lead to model prediction bias because the model tends to minimize the loss function by focusing on the majority class and overlooking the minority class. The datasets for Utilities A and B are moderately imbalanced, with only 15% and 30% of pipe samples experiencing infiltration and 30% and 20% of pipe samples experiencing structural defects, respectively. For these data, the majority class is the set of data for which there is no evidence of the given defect and the minority class is the set of data for which there is evidence of the defect. Three methods for addressing such imbalanced data are applied: (i) the cost‐sensitive learning method; (ii) the synthetic minority oversampling technique (SMOTE) (Bowyer et al. 2002) which is applied for models that are strictly numerical, for example, RF and XGBoost, or its extension, the synthetic minority oversampling technique for nominal and continuous features (SMOTE‐NC), which is applied for models that accommodate categorical and numerical data, for example, LightGBM and CatBoost; and (iii) random undersampling (RUS) method (see also, Fernández et al. 2018 for an extensive discussion of these and other methods for addressing imbalanced data).

The objective of the cost‐sensitive learning method is to train a model to place a heavier weight on the class that has a higher cost in terms of the loss function; that is, for binary models, a higher weight is placed on false predictions of the minority class than on false predictions of the majority class. For the algorithms applied herein, these weights are considered hyperparameters, and the weight of the minority class (i.e., exhibiting a given defect) relative to that of the majority class is the ratio of the number of majority data points and the number of minority data points.

SMOTE, SMOTE‐NC, and RUS require modifying training data to balance the class distribution prior to training and tuning the model. SMOTE and SMOTE‐NC are oversampling methods in which the minority class size is made equal to the majority class size by creating new minority class data points through interpolation of data points between near neighbors. RUS is an undersampling method in which some majority class data points are randomly eliminated to balance the size of the classes. While the dataset may be considered to be small, we explore RUS herein for completeness. The *imblearn.over_sampling.SMOTE* and *imblearn.under_sampling.RandomUnderSampler* API in Python are used to balance the class distribution.

#### 2.3.3 Model Training and Hyperparameter Tuning

The model settings, referred to as hyperparameters, control how the machine processes the data. Each of the algorithms has different hyperparameters, and for each model, the best values of the hyperparameters are determined in the tuning process. To tune the models, a systematic grid search procedure is employed for selecting combinations of hyperparameters and their values that have the highest resulting goodness of fit or so‐called model validation score. For a given model, for each possible hyperparameter‐value combination, training and validation are undertaken with a repeated stratified k‐fold cross‐validation process (see, e.g., the application of k‐fold cross‐validation in Tavakoli et al. 2020). This is executed by partitioning the training data into *k* subsets, undertaking *k* stratified split training and validation processes, and repeating this a number of times; in our study, the number of folds is 4 (*k* = 4) and the number of repetitions is 5. For each of the four training‐validation processes, the model is trained on three of the data subsets and validated on the remaining one, where each subset is used to validate the model one time. The entire training dataset is then shuffled, another partitioning of four subsets is created, and the aforementioned process is repeated four more times. The average of the validation scores from these 20 (4 folds × 5 repetitions) training‐validation processes is the overall validation score for the given model and the given hyperparameter‐value combination. For each model, the hyperparameter‐value combination with the best overall validation score is selected as the settings of the model. The list of hyperparameter‐value combinations for each of the models is provided in Table S1 in the Supporting Information. The *GridSearchCV* and *RepeatedStratifiedKFold* from the *sklearn.model_selection* module in Python are utilized for the grid search cross‐validation and the five repeated fourfold cross‐validation, respectively, in the hyperparameter‐value selection process. Furthermore, the grid search cross‐validation algorithm is configured to run in parallel utilizing “*n*” number of cores in the system, where “*n*” = 16 and resulting in a nearly 16‐fold reduction in processing time. The computational savings are verified on a desktop computer with 13th Gen Intel(R) Core (TM) i7‐13700KF CPU.

#### 2.3.4 Model Evaluation Metrics

The overall performance of each classification model is evaluated using the areas under the ROC curve, denoted as AUC‐ROC, and the PR curve, denoted as AUC‐PR. The ROC curve is considered to be robust to changes in the class distribution (Fawcett 2006), while the PR better represents the performance of models of imbalanced data (Davis and Goadrich 2006). The AUC‐ROC is also used to evaluate the validation score in the hyperparameter tuning process.

The ROC curve represents the trade‐off between the true positive rate (*TPR*) and false positive rate (*FPR*) at various probability thresholds. The probability threshold is the likelihood level at which an identified defect is considered to be present. For example, if the probability threshold is set at 30%, the model will identify a defect as being present if it has a probability of occurrence of at least 30%. *TPR* (Equation (1)) is the portion of pipes with a given defect, in which the given defect is correctly identified, and the *FPR* (Equation (2)) is the portion of pipes that do not exhibit the defect, incorrectly being identified as having the defect. These are defined as follows:

(1) $$\mathrm{TPR}=\frac{\mathrm{TP}}{\mathrm{TP}+\mathrm{FN}}$$

(2) $$\mathrm{FPR}=\frac{\mathrm{FP}}{\mathrm{FP}+\mathrm{TN}}$$

where *TP* is true positive, that is, the number of pipes with a given defect that are correctly identified as having the defect; *FN* is false negative, that is, the number of pipes with a given defect that are incorrectly classified as not having the defect;

The PR curve represents a trade‐off between precision and recall at various probability thresholds. Recall is equivalent to the *TPR* (Equation (1)). Precision (Equation (3)) is the portion of pipes that are correctly classified as having a given defect among the total number of pipes identified as having the given defect. Precision is defined as follows:

(3) $$\text{Precision}=\frac{\mathrm{TP}}{\mathrm{TP}+\mathrm{FP}}$$

The magnitude of AUC‐ROC or AUC‐PR may be used to compare different classification models and to evaluate whether any model performs better than a random classification. The ROC curve for random classification is a diagonal line between point (0,0) and point (1,1), that is, the random classification baseline, where its area is 0.5. Thus, any models with an AUC‐ROC better than 0.5 would perform better than a random classification. The PR curve for random classification is represented by the precision level at the point where recall is one, that is, where all samples are classified has having defects. The random classification baseline, and the area under the baseline, for the PR curve, varies with the distribution of the minority and majority classes (Saito and Rehmsmeier 2015).

Fawcett (2006) asserts that in order to compare performance of two or more models, using the ROC curve, multiple realizations of the data should be evaluated to consider the variance about the curves, to determine whether the models are indeed different. For this study, the relative model performance is evaluated based on 20 subsets of testing data for each model, generated by executing five repeated stratified fourfold cross‐validation on the original testing data. To identify the mean and variance of the ROC curve for each model, a threshold averaging method is used, where *TPR* and *FPR* points are averaged, for a set of probability thresholds (see Fawcett 2006). The Mann–Whitney *U* test, a nonparametric statistical test, is used to compare any two sets of AUC‐ROC generated for any two classification models. The distribution of AUC‐ROC between the two models is found to be different if the *p*‐value, a probability value that describes how likely they are to be the same, is less than or equal to a specified significance level, for example, 0.05 in this study. If the models are considered to be different, the U statistic, which indicates the general magnitude of the difference between two AUC‐ROC, may be used to determine the relative performance of the models, that is, the model with the larger U statistic is considered to be the better model. The same process is applied in this work, when comparing the AUC‐PR between any two models.

ROC curves and PR curves are computed using the *roc_curve* and *precision_recall_curve* functions from the *sklearn.metrics* module in Python. AUC‐ROC and AUC‐PR are computed using *auc* functions from the same module. The Mann–Whitney *U* test is computed using *mannwhitneyu* from the *scipy.stats* module in Python.

#### 2.3.5 SHAP Analysis and Partial Dependence Plots

SHAP, a class additive feature attribution method based on the coalitional game theory, is used to determine the model predictor variable importance. SHAP is applied on the entire dataset (i.e., training and testing) to estimate the degree to which each datapoint contributes to the model target variable value, where positive SHAP values have positive influences on, that is, cause an increase in, the target variable value and negative SHAP values have negative influences. The order of importance of predictor variables is determined based on the mean of the absolute SHAP values. SHAP dependence plots display the relationship between the model predictor variable values and the SHAP values. Partial dependence plots are used to show the average effect of the locational coordinates, the model predictor variables that correspond to the *x*‐ and *y*‐coordinates, on the model target variable, to provide the model‐predicted probability of the given defect occurring at any location within the utility (Friedman 2001). The Python package *shap* and the *sklearn.inspection. PartialDependenceDisplay* API in Python are used for this analysis.

## 3 Results and Discussion

### 3.1 Classification Model Comparison

For each utility (2), target variable (2), algorithm (4), and method for addressing imbalanced data (3), the mean and standard deviation of the AUC‐ROC and AUC‐PR are reported in Tables S2 and S3 (48 = 2 × 2 × 4 × 3 models in total for each performance measure). For a given utility, target variable, and algorithm, the Mann–Whitney *U* test is applied to evaluate whether models based on different methods for addressing imbalanced data are significantly different, and these significantly different cases are highlighted in yellow in Tables S2 and S3. For such cases, the *p*‐values and U statistics, as well as the rank ordered performance based on these, are provided in Tables S2 and S3. For these cases, for a given utility, target variable, and algorithm, the best methods for addressing class imbalance are summarized in Table S4. Based on comparisons of AUC‐ROC, models using the cost‐sensitive learning method are not significantly different from those using SMOTE or SMOTE‐NC; however, they all outperform RUS, where SMOTE variations generally outperform cost‐sensitive learning in terms of their relative superiority to RUS. Comparisons in terms of AUC‐PR indicate that the cost‐sensitive learning and SMOTE or SMOTE‐NC methods perform equally well but outperform RUS. Note that for the structural defects models for Utility B, no one method for addressing imbalanced data outperforms the others.

Since there is no statistical difference between cost‐sensitive learning and SMOTE or SMOTE‐NC methods, the ML‐based models with the highest means of the AUC‐ROC and AUC‐PR are used to compare the performance between the four algorithms. The means of the AUC‐ROC and AUC‐PR of these models and their corresponding standard deviations are provided in Table 4. Based on AUC‐ROC, for both utilities, RF‐based models perform well for classifying infiltration, and LightGBM‐based models perform well for classifying structural defects. Based on AUC‐PR, for Utility A, RF‐ and CatBoost‐based models have the highest mean AUC‐PR, when classifying infiltration and structural defects, respectively. For Utility B, XGBoost‐ and LightGBM‐based models have the highest mean AUC‐PR, when classifying infiltration and structural defects, respectively. However, among all of the models provided in Table 4, only two of the models (i.e., based on only two of the algorithms) for classifying structural defects for Utility B are found to be statistically different from one another.

**TABLE 4:** Models with the highest mean AUC‐ROC and AUC‐PR for each algorithm.

Classification model	Highest mean AUC‐ROC ± 95% CI^a^				Highest mean AUC‐PR ± 95% CI^b^			
	RF	XG‐Boost	Light‐GBM	Cat‐Boost	RF	XG‐Boost	Light‐GBM	Cat‐Boost
Utility A‐I	0.80 ± 0.06^c^	0.77 ± 0.06^d^	0.77 ± 0.05^c^	0.75 ± 0.07^c^	0.41 ± 0.14^d^	0.40 ± 0.15^d^	0.39 ± 0.13^c^	0.38 ± 0.17^d^
Utility A‐SD	0.71 ± 0.07^d^	0.71 ± 0.07^d^	0.72 ± 0.07^d^	0.71 ± 0.06^c^	0.50 ± 0.12^b^	0.49 ± 0.10^c^	0.50 ± 0.11^c^	0.51 ± 0.11^d^
Utility B‐I	0.82 ± 0.07^c^	0.81 ± 0.07^c^	0.81 ± 0.06^c^	0.80 ± 0.07^c^	0.61 ± 0.11^d^	0.63 ± 0.13^d^	0.62 ± 0.12^d^	0.63 ± 0.13^d^
Utility B‐SD	0.71 ± 0.10^c^	0.72 ± 0.10^d^	0.73 ± 0.10^d^	0.71 ± 0.10^d^	0.45 ± 0.16^d^	0.50 ± 0.17^d^	0.51 ± 0.16^d^	0.48 ± 0.15^d^

Abbreviations: I, infiltration; SD, structural defects.

^a^ Mean AUC‐ROC and corresponding standard deviation for a 95% confidence interval, for 20 subsets of test data.

^b^ Mean AUC‐PR and corresponding standard deviation for a 95% confidence interval, for 20 subsets of test data.

^c^ The model trained with SMOTE in the case of RF and XGBoost or SMOTE‐NC in the case of LightGBM and CatBoost to address imbalanced data.

^d^ The model trained with cost‐sensitive learning approach to address imbalanced data.

The summary of the *p*‐values and U statistics for the Utility B structural defects models with the highest mean AUC‐ROC and AUC‐PR is provided in Table 5; the statistics are evaluated for comparison of Utility B structural defects models based on the algorithm in column one, with those based on algorithms listed in the remaining columns. For the Utility B structural defects models, the only statistically significant comparison is between those based on the RF and LightGBM algorithms, with a *p*‐value of 0.03 (i.e., less than 0.05) shown in bold in the table. Based on both AUC‐ROC and AUC‐PR, the U statistic for the LightGBM‐based model compared with the RF‐based model is 281 and is higher than that of the RF‐based model compared with the LightGBM‐based model, which is 119. Because XGBoost‐ and CatBoost‐based models are not statistically different from LightGBM‐ or RF‐based models, we rank the LightGBM‐ and RF‐based models as being in the first and fourth places, respectively. Models based on XGBoost and CatBoost can be ranked based on their U statistics relative to one another. XGBoost‐ and CatBoost‐based models are ranked the second and third, because, based on AUC‐ROC and AUC‐PR, the U statistics for the XGBoost‐based model compared with the CatBoost‐based model are 228 and 219 and are higher than that of the CatBoost‐based model compared with the XGBoost‐based model, which are 173 and 181. It is important to note that the ranking of models in terms of AUC‐ROC and AUC‐PR does not necessarily have to be the same. Tables S5–S7 provide analogous results for the remaining utility‐target variable based models.

**TABLE 5:** Summary of Mann–Whitney tests for the Utility B structural defects models with the highest mean AUC‐ROC and AUC‐PR.

Algorithm (rank)	*p*‐values/U statistics for AUC‐ROC				*p*‐values/U statistics for AUC‐PR			
	RF	XG‐Boost	Light‐GBM	Cat‐Boost	RF	XG‐Boost	Light‐GBM	Cat‐Boost
RF (4)	N/A	0.06/126	**0.03/119**	0.26/158	N/A	0.13/144	**0.03/119**	0.21/153
XGBoost (2)	0.06/271	N/A	0.67/184	0.47/228	0.13/256	N/A	0.52/176	0.62/219
LightGBM (1)	**0.03/281**	0.67/216	N/A	0.27/242	**0.03/281**	0.52/224	N/A	0.16/252
CatBoost (3)	0.26/242	0.47/173	0.27/159	N/A	0.21/247	0.62/181	0.16/148	N/A

*Note:* For Utility B structural defects models, the only statistically significant comparison is between those based on the RF and LightGBM algorithms, with a *p*‐value of 0.03 (i.e., less than 0.05) shown in bold in the table.

**FIGURE 1:**
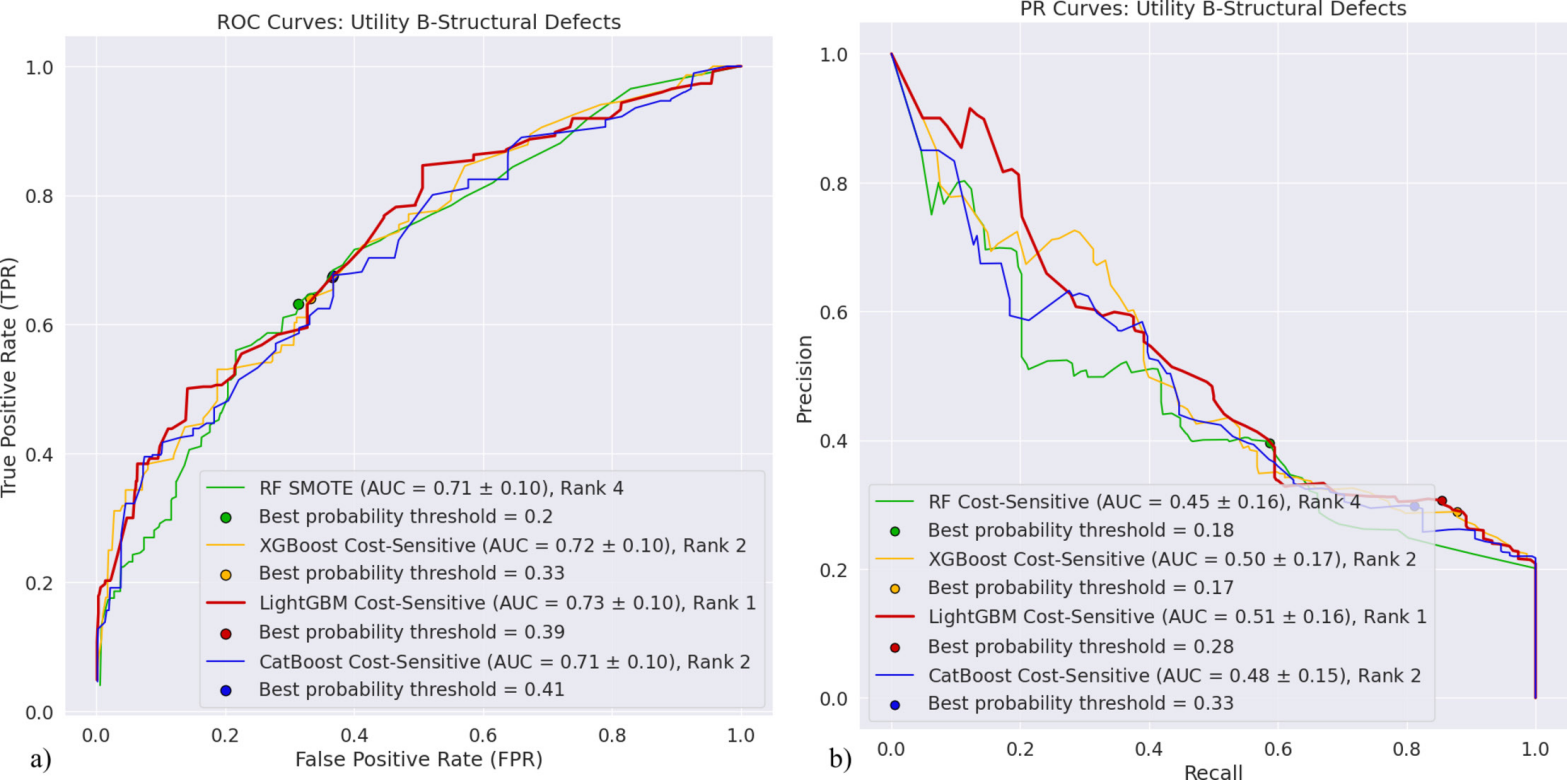
(a) ROC and (b) PR curves for Utility B structural defects classification models with the highest means AUC‐ROC and AUC‐PR for each algorithm.

Only Utility B structural defects classification models may be ranked relative to one another. Thus, Figure 1 provides the ROC and PR curves for these models. The analogous curves for all other models are provided in Figures S1–S3. The curves in Figure 1 are close and cross one another several times; however, based on the Mann–Whitney *U* tests, those for RF‐ and LightGBM‐based models are significantly different from one another. While it appears that the LightGBM‐based model has the highest overall mean AUC‐ROC and AUC‐PR, depending on the preferred level of precision desired, different models and their respective probability threshold levels at the precision value may be preferred. The points on the graph correspond to the *FPR* and *TPR* on the ROC curves or recall and precision values on the PR curves that are the nearest distance to the ideal classification model points, that is, point (0,1) and point (1,1), for ROC and PR curves, respectively. For the given model, these points correspond to the best probability threshold, that is, the probability threshold that yields the best trade‐off between the two values, that is, *FPR* and *TPR* for ROC curves and recall and precision for PR curves. For each algorithm, the best probability threshold for the ROC curve is different from that for the PR curve. The choice of which threshold is preferred depends on the importance placed on the minority class, for example, if one were to place a high penalty on failing to identify the occurrence of a defect, the PR‐based point is important.

**FIGURE 2:**
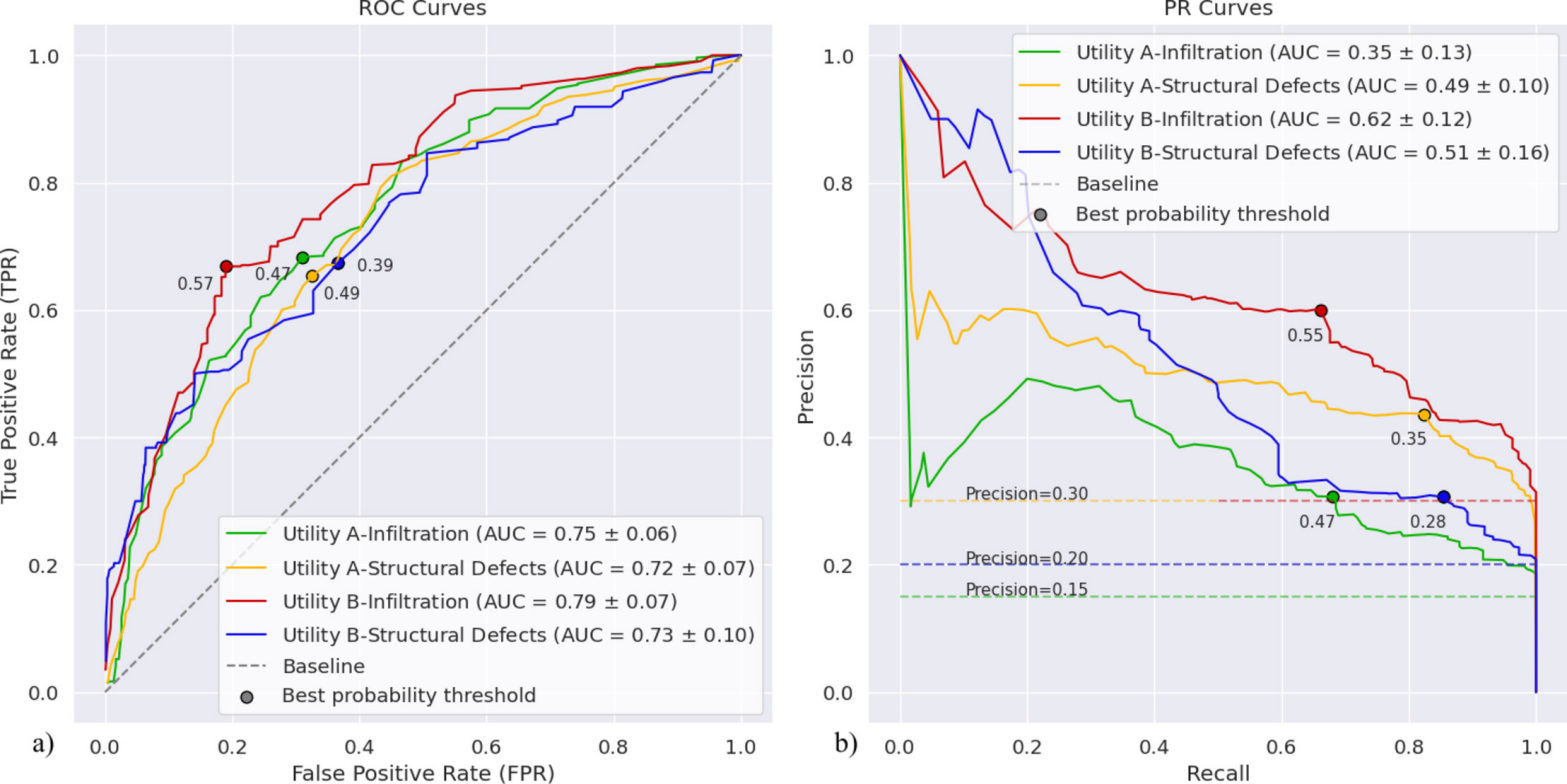
(a) ROC and (b) PR curves for the LightGBM‐based classification models with the cost‐sensitive learning method for a given utility and target variable.

For a visual representation of the influence of class imbalance, the performance of the LightGBM‐based model with the cost‐sensitive learning method for each Utility‐target variable combinations is compared using its ROC curve and the corresponding PR curve in Figure 2. Comparing the ROC and PR curves for these models with the random classification baseline indicates that all models perform better than the random classification. It is important to note that the AUC‐ROC value for random classification is 0.5, and the AUC‐PR value for random classification is based on the distribution of the minority and majority classes. The effect of imbalanced data on the model performance is more prominent on the PR curves. The infiltration classification model for Utility A, trained with the most imbalanced data (approximately 15% minority class), has the lowest AUC‐PR of all four algorithm‐based models. However, this still has an AUC of 0.35 that is 2.33 times larger than that for random classification which has an AUC of 0.15. The structural defects classification models for Utility A and infiltration classification models for Utility B have the same amount of class imbalance (approximately 30% minority class). However, the former model has the second lowest AUC‐PR due to its poor early retrieval, that is, early severely low precision. The model has an AUC‐PR that is 1.63 times larger than that for random classification. In contrast, the latter model has the best overall PR curve and AUC‐PR among all models and performs two times more effectively than the random classification. The structural defects classification model for Utility B (approximately 20% minority class) structural shows good early retrieval, which has a high precision when the recall is small. The model performs 2.55 times better than the random classification.

### 3.2 Predictor Variable Importance and Partial Dependence Plot of the Best Models

To evaluate each model predictor variable in terms of its importance for each classification model, a SHAP analysis was conducted. The mean absolute SHAP values may be used to determine the order of importances of the model predictor variables and their contribution to the target variable, relative to other model predictor variables in the same model. Regarding the location model predictor variables, the SHAP values of the *x*‐ and *y*‐coordinates of the pipe centroid are grouped together (according to the *shap* Python package) to determine the overall effect of pipe location on the model prediction. Their SHAP values are added together for each datapoint, and their summation is treated as one variable. For Utility A‐based models, the pipe shape variable is not considered as all pipes have circular shapes. According to the mean absolute SHAP values from all 12 (four algorithms and three methods for addressing class imbalance) Utility A infiltration classification models, pipe location is the most important variable on average, followed by pipe material, age, length, and size. For Utility A, based on all 12 structural defect classification models, pipe location is the most important variable, followed by material and age, which are jointly ranked second, length, and size. For Utility B, infiltration classification models, pipe age is ranked first, followed by size, location, length, material, and shape. For Utility B, structural defect classification models, age is ranked first, followed by length, location, size, material, and shape. While age and location, indicating environmental effects, are obvious predictors of sewer degradation, the importance of size and length is greater for Utility B than for Utility A because the range of these variables is greater for Utility B. The importance of material is greater for Utility A than for Utility B. Pipe shape does not play a large role in terms of the model prediction for Utility B. The order of importance of the predictor variables for SHAP analyses of each model can be varied and is summarized in Table S8. Figures S4 and S5 show the SHAP summary plots for Utilities A and B, respectively, obtained from LightGBM‐based models with the cost‐sensitive learning method for addressing class imbalance.

**FIGURE 3:**
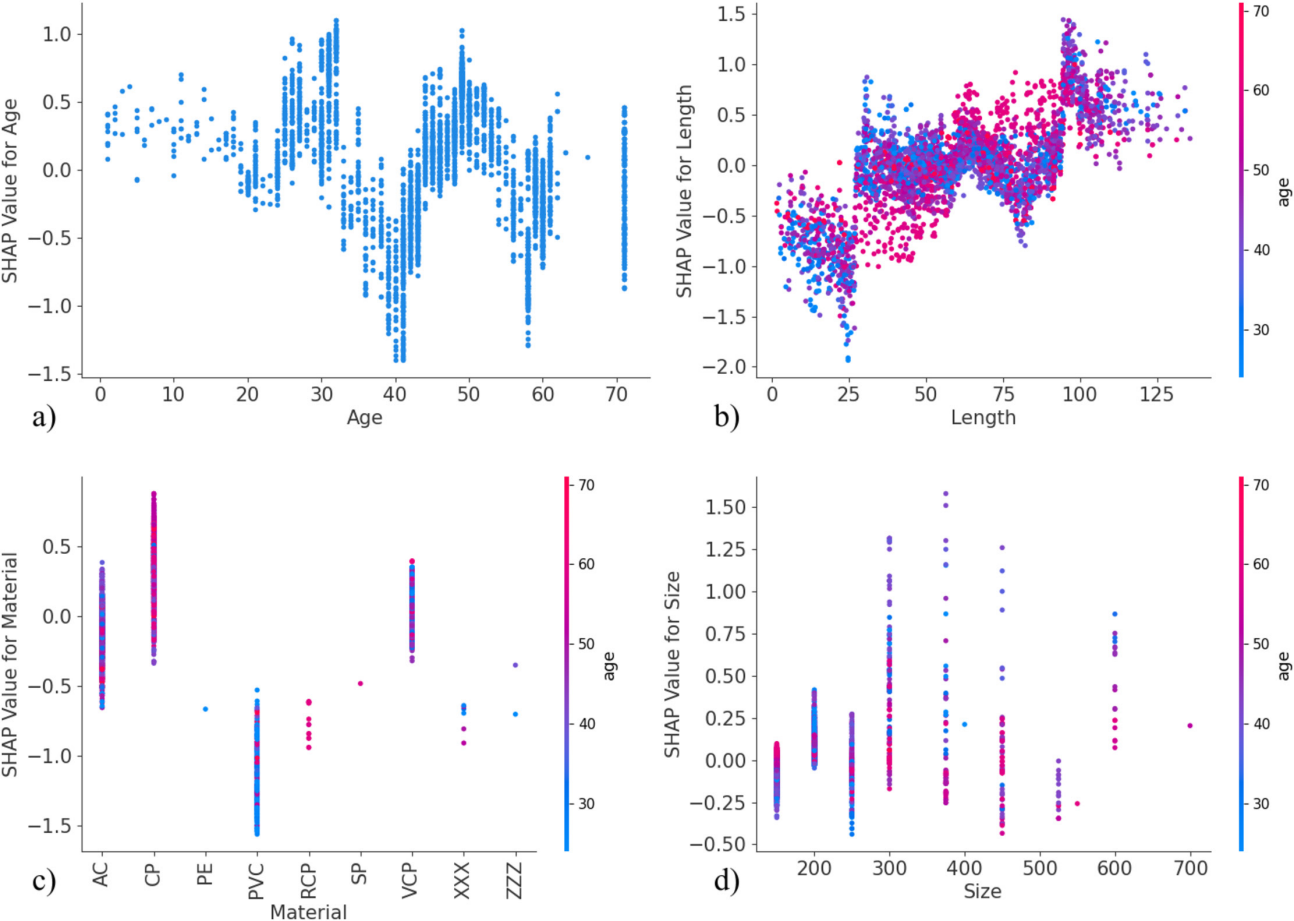
SHAP dependence plots for Utility A infiltration classification model for (a) pipe age, (b) length, (c) material, and (d) size.

**FIGURE 4:**
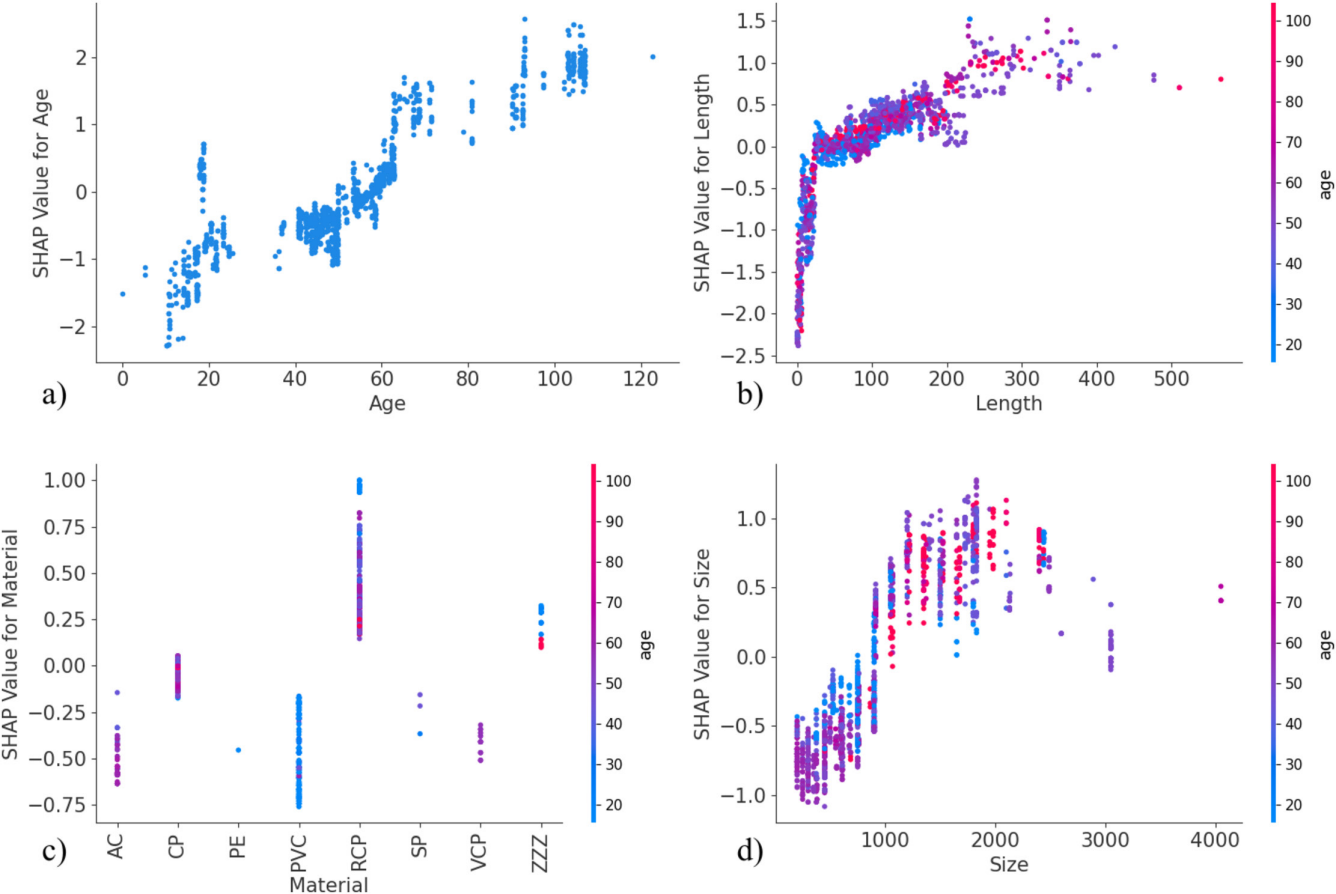
SHAP dependence plots for Utility B infiltration classification model for (a) pipe age, (b) length, (c) material, and (d) size.

All figures from now on are the results of LightGBM‐based models with the cost‐sensitive learning method for addressing class imbalance. Figures 3a–d and 4a–d are SHAP dependence plots of pipe age (a), length (b), material (c), and size (d) for infiltration for Utilities A and B, respectively. Figures S6a–d and S7a–d are similar SHAP dependence plots for structural defects for Utilities A and B, respectively. They explore the influence of different values of the model predictor variables on the target variables, the existence of infiltration, and structural defects. The data points in the figures are colored according to the pipe age to observe the influence of the pipe age, one of the top two model predictor variables, and any correlation of pipe age with the given predictor variable of length, material, and size. The range of SHAP values for each predictor variable in the SHAP summary plots is the same as the SHAP values in their corresponding SHAP dependence plots as shown in Figures S4 and S5. The SHAP dependence plots of the pipe shape are omitted because it has little to no influence on the model prediction as shown in Figures S4 and S5. Even though different models have different rankings of model predictor variables, the SHAP dependence plots from each model are similar.

The SHAP dependence plot of the pipe age in Figure 3a suggests that the infiltration is more likely to happen from ages 0 to 30 years, as indicated by the positive SHAP values. The SHAP value shows a varied trend from ages 30 to 70 years, where an upward trend indicates that the chance of infiltration increases within the given age range and a downward trend indicates that the chance of infiltration decreases. This may be a function of the construction practices at the time of installation of these pipes. Pipes that are poorly installed may show early signs of infiltration where the remaining well‐installed pipes may resist infiltration for the life of the pipe. The relationship between the infiltration and the pipe length is positive‐linear as shown in Figure 3b. The longer the pipe is, the more likely the occurrence of infiltration. Figure 3c shows that a majority of concrete (CP) and vitrified clay (VCP) pipes have positive SHAP values, which lead to an increase in infiltration for Utility A, and a majority of asbestos cement (AC) and polyvinyl chloride (PVC) pipes have a negative effect on, leading to a decrease in, infiltration. In Figure 3d, the majority of data points have SHAP values closer to zero indicating the minimal importance of size variable for effecting the infiltration. It should be noted that one may not find this relationship in other utilities.

The SHAP values in Figure S6a suggest that some structural defects are likely to occur at age 20 years, as indicated by positive SHAP values. The SHAP values decrease from ages 20 to 40 years and increase from ages 40 to 70 years, and indicate that structural defects are more likely to happen at 20 years of pipe life and after approximately 45 years, for Utility A. This phenomenon has been observed in other studies that note construction practices and design flaws may contribute to unexpected early failure (Malek Mohammadi et al. 2020). Figure S6b shows structural defects are likely to occur in the longer pipes than in the shorter pipes. Similar to infiltration, Figure S6c shows that AC and PVC pipes have lesser contribution to structural defects than CP and VCP pipes. Figure S6d shows that the majority of data points have negative SHAP values as pipe size increases indicating that structural defects are less likely to occur in larger pipes than in smaller pipes.

Figure 4a,b shows that SHAP values increase as pipe age and length increase indicating that older and longer pipes are more likely to have infiltration than newer and shorter pipes for Utility B. Figure 4c shows that AC, CP, PVC, and VCP pipes have a negative effect on infiltration, while reinforced concrete (RCP) and other material (ZZZ) pipes have a positive effect on infiltration. There is only a single data point for polyethylene (PE) and three data points for steel (SP) pipes. Thus, they are not used in the analysis. In addition, most of the PVC pipes are less than 40 years old indicating that the pipe age may influence the PVC pipe SHAP values. However, this does not apply for other types of pipe material. Figure 4d shows that SHAP values have an upward trend for pipe sizes increasing from 200 to 1700 mm and the downward trend from 1700 to 3000 mm. However, most pipes from 1000 mm onwards have positive SHAP values indicating that they are likely to have infiltration.

As shown in Figure S7, an upward trend of SHAP values as the variable values increase is observed for the pipe age, length, and size for structural defects for Utility B. In addition, AC, CP, and PVC pipes have a lower likelihood of having structural defects, while RCP, VCP, and other pipes have a positive effect on structural defects. Lastly, for each classification model, there is no clear indication that shows that pipe age influences the other three model predictor variable SHAP values, as the color distribution of the data points does not follow the pattern of the pipe age SHAP values.

**FIGURE 5:**
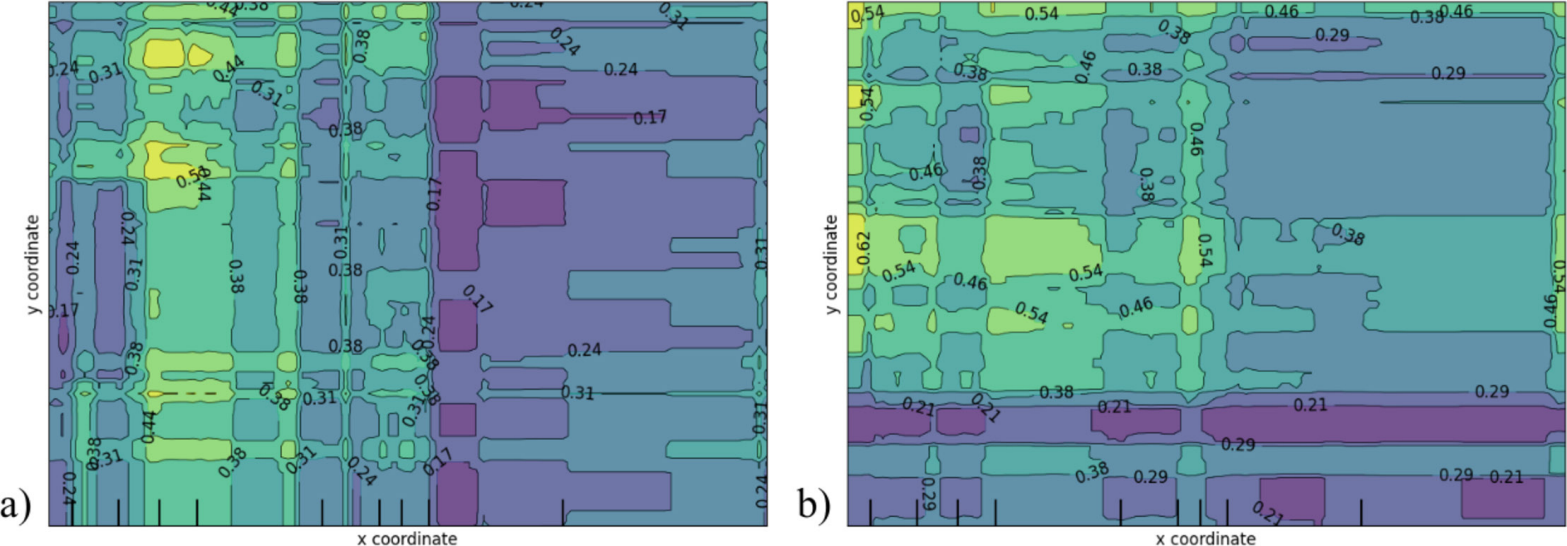
Partial dependence plot between *x*‐ and *y*‐coordinates, likelihood of (a) infiltration and (b) structural defects in Utility A where lighter colors represent a larger likelihood while darker colors suggest a lower likelihood.

SHAP partial dependence plots, provided in Figure 5, show the average effect of the locational coordinates (i.e., *x*‐ and *y*‐ coordinates variables) on the predictions of infiltration (a) and structural defects (b) for Utility A. The average predicted probability that infiltration and structural defects are found in Utility A is denoted, where the darker the color, the lower the probability. Here, average predicted probability of infiltration is to be found in pipes located on the western side of Utility A, while structural defects are to be found in pipes located in the upper portion of the western side. One cannot divide Utility B into distinct infiltration and structural area cluster; nonetheless, partial dependence plots for Utility B are provided in Figure S8.

## 4 Conclusions

Unlike conventional sewer deterioration models that rely on subjective interpretation of condition, this paper develops and evaluates ML‐based binary classification models that identify the expected occurrence of specific defects of concern to utilities, using CCTV information as well as other observations from the field. The results from the models can assist with the pipe inspection scheduling by generating the inventory of pipes that are likely to have specific defects, as well as identifying the areas within the network where these specific defects are more likely to occur and should be scheduled for inspection and repair.

The models are capable of accommodating data from various condition assessment standards, as the classification procedure requires only the grouping of defects with similar characteristics rather than pipe condition scores that could have different ranking criteria and scaling methods for different condition assessment standards. Four algorithms are examined, two of which can accommodate categorical variables innately, and three methods for accommodating class imbalances. New methods for evaluating these models that account for uncertainty are employed to distinguish the model performance, and SHAP analysis is used to identify important predictor variables.

The models are trained and tested on data from two utilities to identify infiltration and structural defects within their network. AUC‐ROC and AUC‐PR were used to evaluate the performance of each model and indicate that there is no statistical difference between most models, except for one case where a LightGBM‐based model outperforms an RF‐based model. However, if additional categorical predictor variables are included in the future, it may be preferable to use an algorithm that can innately handle categorical variables, rather than relying on techniques such as one‐hot encoding, which create multiple binary features, can increase feature dimensionality, and may lead to feature sparsity. In most cases, models that employ cost‐sensitive learning and SMOTE methods outperform models that apply RUS. One drawback of RUS for this study is that it decreases the data size, though RUS may be more suitable for larger datasets. The performance of the models is affected by imbalanced data, and such data‐based models can be evaluated via PR curves. All novel classification models are proven to perform up to 2.5 times better than random classification.

The SHAP analysis shows that pipe age and location are two of the most influential variables for infiltration and structural defects in both utilities. Pipe size and length have more influence for Utility B, which has a larger sewer system, than for Utility A, and pipe material has more influence for Utility A than for Utility B. The SHAP dependence plots show that PVC pipes have the least influence on the infiltration and structural defects for both utilities in comparison with other pipe materials, although the SHAP analysis indicates that pipe material has a lower predictor variable importance ranking for Utility B.

The methodology developed and demonstrated in this paper provides a general approach for identifying other sewer defects in these and other utility settings. Environmental model predictor variables, such as soil conditions and surface loads, as well as road types and proximity to critical infrastructure, could be integrated as model predictor variables to improve model performance and applicability. Models such as those developed herein may also be incorporated in likelihood of failure and consequence of failure analyses, and key features identified with SHAP analysis provide information on which utilities may focus to undertake asset management of the network and avoid premature sewer failure.

## Author Contributions

**Vannary Seng:** data curation, methodology, visualization, writing – original draft, writing review and editing, software, formal analysis. **Barbara J. Lence:** supervision, funding acquisition, writing – review and editing, project administration, formal analysis. **Sudhir Kshirsagar:** conceptualization, methodology, software, supervision, funding acquisition, project administration, validation. **Srujana Rangapuram:** software, validation. **Pavan Saranguhewa:** software, validation.

## Conflicts of Interest

The authors declare no conflicts of interest.

## Supporting information


**TABLE S1:** List of hyperparameter values used during cross‐validation grid search.
**TABLE S2:** The average AUC‐ROC of each classification model and its U statistics, p‐value, and rank when comparing its performance with model with other imbalanced data managing techniques, as determined by the Mann–Whitney U test at significance level equals to 0.05.
**TABLE S3:** The area under the average PR curves of each classification model and its U statistics, p‐value, and rank when comparing its performance with model with other imbalanced data managing techniques, as determined by the Mann–Whitney U test at significance level equals to 0.05.
**TABLE S4:** Best methods to address imbalanced data for each utility‐target variable‐algorithm‐based model.
**TABLE S5:** Summary of Mann–Whitney U tests for the Utility A infiltration models with the highest mean AUC‐ROC and AUC‐PR.
**TABLE S6:** Summary of Mann–Whitney U tests for the Utility A structural defects models with the highest mean AUC‐ROC and AUC‐PR.
**TABLE S7:** Summary of Mann–Whitney U tests for the Utility B infiltration models with the highest mean AUC‐ROC and AUC‐PR.
**FIGURE S1:** (a) ROC and (b) PR curves of Utility A infiltration classification models with the highest means AUC‐ROC and AUC‐PR for each algorithm.
**FIGURE S2:** (a) ROC and (b) PR curves of Utility A structural defects classification models with the highest means AUC‐ROC and AUC‐PR for each algorithm.
**FIGURE S3:** (a) ROC and (b) PR curves of Utility B infiltration classification models with the highest means AUC‐ROC and AUC‐PR for each algorithm.
**FIGURE S4:** Model predictor variable importance for (a) infiltration and (b) structural defects classification models for Utility A.
**FIGURE S5:** Model predictor variable importance for (a) infiltration and (b) structural defects classification models for Utility B.
**TABLE S8:** Rank ordered model predictor variables from first to last place of each classification model.
**FIGURE S6:** SHAP dependence plots for Utility A structural defects classification model for (a) pipe age, (b) length, (c) material, and (d) size.
**FIGURE S7:** SHAP dependence plots for Utility B structural defects classification model for (a) pipe age, (b) length, (c) material, and (d) size.
**Figure S8:** Partial dependence plot for x‐ and y‐ coordinates, likelihood of (a) infiltration and (b) structural defects for Utility B where lighter colors represent a larger likelihood while darker colors suggest a lower likelihood.

## Data Availability

The data that support the findings of this study are available on request from the corresponding author. The data are not publicly available due to privacy or ethical restrictions.
