# Supplementary material for: Novel Sewer Defect Prediction Leveraging Advanced Machine Learning (ML) Models
Source: Water Environ Res. 2026 Mar 19;98(3):e70338. doi: 10.1002/wer.70338 (PMC13001702; doi:10.1002/wer.70338)
Supplement: Supplementary file 1 — TABLE S1: List of hyperparameter values used during cross‐validation grid search. TABLE S2: The average AUC‐ROC of each classification model and its U statistics, p‐value, and rank when comparing its performance with model with other imbalanced data managing techniques, as determined by the Mann–Whitney U test at significance level equals to 0.05. TABLE S3: The area under the average PR curves of each classification model and its U statistics, p‐value, and rank when comparing its performance with model with other imbalanced data managing techniques, as determined by the Mann–Whitney U test at significance level equals to 0.05. TABLE S4: Best methods to address imbalanced data for each utility‐target variable‐algorithm‐based model. TABLE S5: Summary of Mann–Whitney U tests for the Utility A infiltration models with the highest mean AUC‐ROC and AUC‐PR. TABLE S6: Summary of Mann–Whitney U tests for the Utility A structural defects models with the highest mean AUC‐ROC and AUC‐PR. TABLE S7: Summary of Mann–Whitney U tests for the Utility B infiltration models with the highest mean AUC‐ROC and AUC‐PR. FIGURE S1: (a) ROC and (b) PR curves of Utility A infiltration classification models with the highest means AUC‐ROC and AUC‐PR for each algorithm. FIGURE S2: (a) ROC and (b) PR curves of Utility A structural defects classification models with the highest means AUC‐ROC and AUC‐PR for each algorithm. FIGURE S3: (a) ROC and (b) PR curves of Utility B infiltration classification models with the highest means AUC‐ROC and AUC‐PR for each algorithm. FIGURE S4: Model predictor variable importance for (a) infiltration and (b) structural defects classification models for Utility A. FIGURE S5: Model predictor variable importance for (a) infiltration and (b) structural defects classification models for Utility B. TABLE S8: Rank ordered model predictor variables from first to last place of each classification model. FIGURE S6: SHAP dependence plots for Utility A structural defects classi [file WER-98-e70338-s001.docx]

**SUPPLEMENTARY MATERIAL**

## Hyperparameter Tuning

Table S1 shows list of hyperparamets and their values used to find the optimal model for each combination of algorithm and method used to address imbalanced data.

TABLE S1. List of hyperparameter values used during cross-validation grid search.

| Parameters | RF | XGBoost | LightGBM | CatBoost |
| --- | --- | --- | --- | --- |
| n_estimators/  iterations | [50, 100, 150, 200] | [50, 100, 150, 200] | [50, 100, 150, 200] | [50, 100, 150, 200] |
| max_features | [None, sqrt, log2] | - | - | - |
| class_weight/  scale_pos_weight | [None, balanced] | majority class to minority class ratio | majority class to minority class ratio | majority class to minority class ratio |
| criterion | [gini, entropy] | - | - | - |
| max_depth/  depth | - | [10, 30, 50, 80, 100] | [10, 30, 50, 80, 100] | [4, 6, 8, 10] |
| learning_rate | - | [0.03, 0.05, 0.1, 0.15, 0.2, 0.25, 0.3] | [0.03, 0.05, 0.1, 0.15, 0.2, 0.25, 0.3] | [0.03, 0.05, 0.1, 0.15, 0.2, 0.25, 0.3] |
| lambda/  lambda_l2/  l2_leaf_reg | - | [0, 1, 3, 9] | [0, 1, 3, 9] | [0, 1, 3, 9] |
| num_leaves | - | - | [10, 30, 50, 80, 100, 200, 300] | - |

## Classification Model Comparison

The distribution of AUC-ROC or AUC-PR between the two models are found to be different if the p-value, a probability value that describes how likely they are to be the same, is less than or equal to a specified significance level, e.g., 0.05 in this study. If the models are considered to be different, the U statistic may be used to determine the relative performance of the models, i.e., the model with the larger U statistic is considered to be the better model. The models may be ranked relative to one another if at least one pair of models is found to be different. In Table S2 and Table S3, models that are considered to be significantly different from one another are highlighted in yellow. For a given utility, target variable, and algorithm, the best methods for addressing class imbalance, are summarized in Table S4.

TABLE S2. The average AUC-ROC of each classification model and its U statistics, p-value, and rank when comparing its performance with model with other imbalanced data managing techniques, as determined by the Mann-Whitney U test at significance level equals to 0.05.

| Utility | Target variable | Algorithm | Imbalanced data managing technique | Mean AUC | Two standard deviations | U Statistics when comparing the model to | | | p-values when comparing the model to | | | Rank |
| --- | --- | --- | --- | --- | --- | --- | --- | --- | --- | --- | --- | --- |
|  |  |  |  |  |  | Cost-sensitive | RUS | SMOTE/ SMOTENC | Cost-sensitive | RUS | SMOTE/ SMOTENC |  |
| Utility A | Infiltration | RF | Cost-Sensitive | 0.80 | 0.06 | - | 270 | 198 | - | 0.06 | 0.97 | 2 |
|  |  |  | RUS | 0.77 | 0.08 | 130 | - | 118 | 0.06 | - | 0.03 | 3 |
|  |  |  | SMOTE | 0.80 | 0.06 | 202 | 282 | - | 0.97 | 0.03 | - | 1 |
|  |  | XGBoost | Cost-Sensitive | 0.77 | 0.06 | - | 304 | 213 | - | 0.01 | 0.74 | 1 |
|  |  |  | RUS | 0.73 | 0.09 | 96 | - | 106 | 0.01 | - | 0.01 | 3 |
|  |  |  | SMOTE | 0.77 | 0.06 | 187 | 294 | - | 0.74 | 0.01 | - | 1 |
|  |  | LightGBM | Cost-Sensitive | 0.75 | 0.06 | - | 226 | 132 | - | 0.49 | 0.07 | 2 |
|  |  |  | RUS | 0.74 | 0.09 | 174 | - | 104 | 0.49 | - | 0.01 | 3 |
|  |  |  | SMOTE-NC | 0.77 | 0.05 | 268 | 296 | - | 0.07 | 0.01 | - | 1 |
|  |  | CatBoost | Cost-Sensitive | 0.75 | 0.09 | - | 205.5 | 169 | - | 0.89 | 0.41 | - |
|  |  |  | RUS | 0.74 | 0.10 | 194.5 | - | 159 | 0.89 | - | 0.27 | - |
|  |  |  | SMOTE-NC | 0.75 | 0.07 | 231 | 241 | - | 0.41 | 0.27 | - | - |
|  | Structural Defects | RF | Cost-Sensitive | 0.71 | 0.07 | - | 226 | 208 | - | 0.49 | 0.84 | - |
|  |  |  | RUS | 0.70 | 0.06 | 174 | - | 163 | 0.49 | - | 0.32 | - |
|  |  |  | SMOTE | 0.71 | 0.07 | 192 | 237 | - | 0.84 | 0.32 | - | - |
|  |  | XGBoost | Cost-Sensitive | 0.71 | 0.07 | - | 248 | 197 | - | 0.20 | 0.95 | - |
|  |  |  | RUS | 0.70 | 0.08 | 152 | - | 146 | 0.20 | - | 0.15 | - |
|  |  |  | SMOTE | 0.71 | 0.06 | 203 | 254 | - | 0.95 | 0.15 | - | - |
|  |  | LightGBM | Cost-Sensitive | 0.72 | 0.07 | - | 287 | 202 | - | 0.02 | 0.97 | 1 |
|  |  |  | RUS | 0.69 | 0.07 | 113 | - | 107 | 0.02 | - | 0.01 | 3 |
|  |  |  | SMOTE-NC | 0.71 | 0.07 | 198 | 293 | - | 0.97 | 0.01 | - | 1 |
|  |  | CatBoost | Cost-Sensitive | 0.71 | 0.07 | - | 254 | 198.5 | - | 0.15 | 0.98 | - |
|  |  |  | RUS | 0.70 | 0.06 | 146 | - | 148 | 0.15 | - | 0.16 | - |
|  |  |  | SMOTE-NC | 0.71 | 0.06 | 201.5 | 252 | - | 0.98 | 0.16 | - | - |
| Utility B | Infiltration | RF | Cost-Sensitive | 0.81 | 0.07 | - | 235 | 166.5 | - | 0.35 | 0.37 | - |
|  |  |  | RUS | 0.80 | 0.07 | 165 | - | 142 | 0.35 | - | 0.12 | - |
|  |  |  | SMOTE | 0.82 | 0.07 | 233.5 | 258 | - | 0.37 | 0.12 | - | - |
|  |  | XGBoost | Cost-Sensitive | 0.80 | 0.08 | - | 248 | 182.5 | - | 0.20 | 0.65 | - |
|  |  |  | RUS | 0.78 | 0.08 | 152 | - | 139 | 0.20 | - | 0.10 | - |
|  |  |  | SMOTE | 0.81 | 0.07 | 217.5 | 261 | - | 0.65 | 0.10 | - | - |
|  |  | LightGBM | Cost-Sensitive | 0.79 | 0.07 | - | 216.5 | 132.5 | - | 0.67 | 0.07 | 2 |
|  |  |  | RUS | 0.78 | 0.09 | 183.5 | - | 119.5 | 0.67 | - | 0.03 | 3 |
|  |  |  | SMOTE-NC | 0.81 | 0.06 | 267.5 | 280.5 | - | 0.07 | 0.03 | - | 1 |
|  |  | CatBoost | Cost-Sensitive | 0.80 | 0.07 | - | 274 | 181.5 | - | 0.05 | 0.63 | 1 |
|  |  |  | RUS | 0.78 | 0.08 | 126 | - | 118 | 0.05 | - | 0.03 | 3 |
|  |  |  | SMOTE-NC | 0.80 | 0.07 | 218.5 | 282 | - | 0.63 | 0.03 | - | 1 |
|  | Structural Defects | RF | Cost-Sensitive | 0.69 | 0.11 | - | 206.5 | 167.5 | - | 0.87 | 0.39 | - |
|  |  |  | RUS | 0.68 | 0.10 | 193.5 | - | 160.5 | 0.87 | - | 0.29 | - |
|  |  |  | SMOTE | 0.71 | 0.10 | 232.5 | 239.5 | - | 0.39 | 0.29 | - | - |
|  |  | XGBoost | Cost-Sensitive | 0.72 | 0.10 | - | 265 | 211 | - | 0.08 | 0.78 | - |
|  |  |  | RUS | 0.69 | 0.09 | 135 | - | 149 | 0.08 | - | 0.17 | - |
|  |  |  | SMOTE | 0.71 | 0.10 | 189 | 251 | - | 0.78 | 0.17 | - | - |
|  |  | LightGBM | Cost-Sensitive | 0.73 | 0.10 | - | 215.5 | 222 | - | 0.68 | 0.56 | - |
|  |  |  | RUS | 0.73 | 0.07 | 184.5 | - | 214 | 0.68 | - | 0.71 | - |
|  |  |  | SMOTE-NC | 0.71 | 0.10 | 178 | 186 | - | 0.56 | 0.71 | - | - |
|  |  | CatBoost | Cost-Sensitive | 0.71 | 0.10 | - | 234 | 250.5 | - | 0.36 | 0.18 | - |
|  |  |  | RUS | 0.69 | 0.11 | 166 | - | 214 | 0.36 | - | 0.71 | - |
|  |  |  | SMOTE-NC | 0.68 | 0.12 | 149.5 | 186 | - | 0.18 | 0.71 | - | - |

TABLE S3. The area under the average PR curves of each classification model and its U statistics, p-value, and rank when comparing its performance with model with other imbalanced data managing techniques, as determined by the Mann-Whitney U test at significance level equals to 0.05.

| Utility | Target variable | Algorithm | Imbalanced data managing technique | Mean AUC | Two standard deviations | U Statistics when comparing the model to | | | p-values when comparing the model to | | | Rank |
| --- | --- | --- | --- | --- | --- | --- | --- | --- | --- | --- | --- | --- |
|  |  |  |  |  |  | Cost-sensitive | RUS | SMOTE/ SMOTE-NC | Cost-sensitive | RUS | SMOTE/ SMOTE-NC |  |
| Utility A | Infiltration | RF | Cost-Sensitive | 0.41 | 0.14 | - | 274 | 216 | - | 0.05 | 0.68 | 1 |
|  |  |  | RUS | 0.36 | 0.13 | 126 | - | 140 | 0.05 | - | 0.11 | 3 |
|  |  |  | SMOTE | 0.40 | 0.13 | 184 | 260 | - | 0.68 | 0.11 | - | 2 |
|  |  | XGBoost | Cost-Sensitive | 0.40 | 0.15 | - | 259 | 265 | - | 0.11 | 0.08 | - |
|  |  |  | RUS | 0.35 | 0.15 | 141 | - | 209 | 0.11 | - | 0.82 | - |
|  |  |  | SMOTE | 0.36 | 0.11 | 135 | 191 | - | 0.08 | 0.82 | - | - |
|  |  | LightGBM | Cost-Sensitive | 0.35 | 0.13 | - | 159 | 136 | - | 0.27 | 0.09 | - |
|  |  |  | RUS | 0.37 | 0.17 | 241 | - | 184 | 0.27 | - | 0.68 | - |
|  |  |  | SMOTE-NC | 0.39 | 0.13 | 264 | 216 | - | 0.09 | 0.68 | - | - |
|  |  | CatBoost | Cost-Sensitive | 0.38 | 0.17 | - | 241 | 212 | - | 0.27 | 0.76 | - |
|  |  |  | RUS | 0.35 | 0.17 | 159 | - | 172 | 0.27 | - | 0.46 | - |
|  |  |  | SMOTE-NC | 0.38 | 0.15 | 188 | 228 | - | 0.76 | 0.46 | - | - |
|  | Structural Defects | RF | Cost-Sensitive | 0.50 | 0.12 | - | 250 | 206 | - | 0.18 | 0.88 | - |
|  |  |  | RUS | 0.48 | 0.09 | 150 | - | 151 | 0.18 | - | 0.19 | - |
|  |  |  | SMOTE | 0.50 | 0.12 | 194 | 249 | - | 0.88 | 0.19 | - | - |
|  |  | XGBoost | Cost-Sensitive | 0.49 | 0.10 | - | 244 | 186 | - | 0.24 | 0.71 | - |
|  |  |  | RUS | 0.47 | 0.09 | 156 | - | 133 | 0.24 | - | 0.07 | - |
|  |  |  | SMOTE | 0.49 | 0.10 | 214 | 267 | - | 0.71 | 0.07 | - | - |
|  |  | LightGBM | Cost-Sensitive | 0.49 | 0.10 | - | 277 | 184 | - | 0.04 | 0.68 | 1 |
|  |  |  | RUS | 0.46 | 0.09 | 123 | - | 119 | 0.04 | - | 0.03 | 3 |
|  |  |  | SMOTE-NC | 0.50 | 0.11 | 216 | 281 | - | 0.68 | 0.03 | - | 1 |
|  |  | CatBoost | Cost-Sensitive | 0.51 | 0.11 | - | 264 | 231 | - | 0.09 | 0.41 | - |
|  |  |  | RUS | 0.48 | 0.09 | 136 | - | 160 | 0.09 | - | 0.29 | - |
|  |  |  | SMOTE-NC | 0.49 | 0.09 | 169 | 240 | - | 0.41 | 0.29 | - | - |
| Utility B | Infiltration | RF | Cost-Sensitive | 0.61 | 0.11 | - | 272 | 256 | - | 0.05 | 0.13 | - |
|  |  |  | RUS | 0.57 | 0.13 | 128 | - | 181 | 0.05 | - | 0.62 | - |
|  |  |  | SMOTE | 0.60 | 0.14 | 144 | 219 | - | 0.13 | 0.62 | - | - |
|  |  | XGBoost | Cost-Sensitive | 0.63 | 0.13 | - | 271 | 271 | - | 0.06 | 0.06 | - |
|  |  |  | RUS | 0.58 | 0.14 | 129 | - | 176 | 0.06 | - | 0.52 | - |
|  |  |  | SMOTE | 0.59 | 0.12 | 129 | 224 | - | 0.06 | 0.52 | - | - |
|  |  | LightGBM | Cost-Sensitive | 0.62 | 0.12 | - | 252 | 235 | - | 0.16 | 0.35 | - |
|  |  |  | RUS | 0.58 | 0.14 | 148 | - | 177 | 0.16 | - | 0.54 | - |
|  |  |  | SMOTE-NC | 0.60 | 0.12 | 165 | 223 | - | 0.35 | 0.54 | - | - |
|  |  | CatBoost | Cost-Sensitive | 0.63 | 0.13 | - | 302 | 232 | - | 0.01 | 0.39 | 1 |
|  |  |  | RUS | 0.54 | 0.15 | 98 | - | 111 | 0.01 | - | 0.02 | 3 |
|  |  |  | SMOTE-NC | 0.61 | 0.11 | 168 | 289 | - | 0.39 | 0.02 | - | 1 |
|  | Structural Defects | RF | Cost-Sensitive | 0.45 | 0.16 | - | 191 | 221 | - | 0.82 | 0.58 | - |
|  |  |  | RUS | 0.46 | 0.15 | 209 | - | 236 | 0.82 | - | 0.34 | - |
|  |  |  | SMOTE | 0.44 | 0.15 | 179 | 164 | - | 0.58 | 0.34 | - | - |
|  |  | XGBoost | Cost-Sensitive | 0.50 | 0.17 | - | 223 | 232 | - | 0.54 | 0.39 | - |
|  |  |  | RUS | 0.47 | 0.16 | 177 | - | 212 | 0.54 | - | 0.76 | - |
|  |  |  | SMOTE | 0.48 | 0.15 | 168 | 188 | - | 0.39 | 0.76 | - | - |
|  |  | LightGBM | Cost-Sensitive | 0.51 | 0.16 | - | 240 | 259 | - | 0.29 | 0.11 | - |
|  |  |  | RUS | 0.48 | 0.15 | 160 | - | 246 | 0.29 | - | 0.22 | - |
|  |  |  | SMOTE-NC | 0.46 | 0.16 | 141 | 154 | - | 0.11 | 0.22 | - | - |
|  |  | CatBoost | Cost-Sensitive | 0.48 | 0.15 | - | 227 | 257 | - | 0.47 | 0.13 | - |
|  |  |  | RUS | 0.47 | 0.15 | 173 | - | 215 | 0.47 | - | 0.69 | - |
|  |  |  | SMOTE-NC | 0.45 | 0.15 | 143 | 185 | - | 0.13 | 0.69 | - | - |

TABLE S4. Best methods to address imbalanced data for each utility-target variable-algorithm-based model.

| Classification model | Based on AUC-ROC | | | | Based on AUC-PR | | | |
| --- | --- | --- | --- | --- | --- | --- | --- | --- |
|  | RF | XG-Boost | Light-GBM | Cat-Boost | RF | XG-Boost | Light-GBM | Cat-Boost |
| Utility A-I | SMOTE | CS/ SMOTE | SMOTE-NC | - | CS | - | - | - |
| Utility A-SD | - | - | CS/ SMOTE-NC | - | - | - | CS/ SMOTE-NC | - |
| Utility B-I | - | - | - | CS/ SMOTE-NC | - | - | - | CS/ SMOTE-NC |
| Utility B-SD | - | - | - | - | - | - | - | - |

Note: I=Infiltration; SD=Structural Defects; CS=Cost-sensitive learning.

The summary of the p-values and U statistics for Utility A infiltration models with the highest mean AUC-ROC and AUC-PR are provided in Table S5, the statistics are evaluated for comparison of Utility A infiltration models based on the algorithm in column one, with those based on algorithms listed in the remaining columns. Tables S6 and S7 provide analogous results for Utility A structural defects and Utility B infiltration models, respectively.

TABLE S5. Summary of Mann-Whitney U Tests for the Utility A infiltration models with the highest mean AUC-ROC and AUC-PR.

| Algorithm | p-values/U statistics for AUC-ROC | | | | p-values/U statistics for AUC-PR | | | |
| --- | --- | --- | --- | --- | --- | --- | --- | --- |
|  | RF | XG-Boost | Light-GBM | Cat-Boost | RF | XG-Boost | Light-GBM | Cat-Boost |
| RF | N/A | 0.52/ 224.5 | 0.76/ 212 | 0.19/ 249 | N/A | 0.92/ 196 | 0.56/ 222 | 0.32/ 237 |
| XGBoost | 0.52/ 175.5 | N/A | 0.76/ 188 | 0.43/ 229.5 | 0.92/ 204 | N/A | 0.68/ 216 | 0.41/ 231 |
| LightGBM | 0.76/ 188 | 0.76/ 212 | N/A | 0.17/ 251 | 0.56/ 178 | 0.68/ 184 | N/A | 0.62/ 219 |
| CatBoost | 0.19/ 151 | 0.43/ 170.5 | 0.17/ 149 | N/A | 0.32/ 163 | 0.41/ 169 | 0.62/ 181 | N/A |

Note: These models are not ranked because no one model is statistically different from one another.

TABLE S6. Summary of Mann-Whitney U Tests for the Utility A structural defects models with the highest mean AUC-ROC and AUC-PR.

| Algorithm | p-values/U statistics for AUC-ROC | | | | p-values/U statistics for AUC-PR | | | |
| --- | --- | --- | --- | --- | --- | --- | --- | --- |
|  | RF | XG-Boost | Light-GBM | Cat-Boost | RF | XG-Boost | Light-GBM | Cat-Boost |
| RF | N/A | 0.47/ 173 | 0.34/ 164 | 0.58/ 179 | N/A | 0.82/ 191 | 0.92/ 196 | 0.46/ 172 |
| XGBoost | 0.47/ 227 | N/A | 0.58/ 179 | 0.95/ 197 | 0.82/ 209 | N/A | 0.84/ 192 | 0.54/ 177 |
| LightGBM | 0.34/ 236 | 0.58/ 221 | N/A | 0.62/ 219 | 0.92/ 204 | 0.84/ 208 | N/A | 0.76/ 188 |
| CatBoost | 0.58/ 221 | 0.95/ 203 | 0.62/ 181 | N/A | 0.46/ 228 | 0.54/ 223 | 0.76/ 212 | N/A |

Note: These models are not ranked because no one model is statistically different from one another.

TABLE S7. Summary of Mann-Whitney U Tests for the Utility B infiltration models with the highest mean AUC-ROC and AUC-PR.

| Algorithm | p-values/U statistics for AUC-ROC | | | | p-values/U statistics for AUC-PR | | | |
| --- | --- | --- | --- | --- | --- | --- | --- | --- |
|  | RF | XG-Boost | Light-GBM | Cat-Boost | RF | XG-Boost | Light-GBM | Cat-Boost |
| RF | N/A | 0.91/ 195.5 | 0.67/ 183.5 | 0.80/ 210 | N/A | 0.44/ 171 | 0.74/ 187 | 0.36/ 166 |
| XGBoost | 0.91/ 204.5 | N/A | 0.76/ 188 | 0.78/ 211 | 0.44/ 229 | N/A | 0.64/ 218 | 0.90/ 195 |
| LightGBM | 0.67/ 216.5 | 0.76/ 212 | N/A | 0.48/ 226.5 | 0.74/ 213 | 0.64/ 182 | N/A | 0.52/ 176 |
| CatBoost | 0.80/ 190 | 0.78/ 189 | 0.48/ 173.5 | N/A | 0.36/ 234 | 0.90/ 205 | 0.52/ 224 | N/A |

Note: These models are not ranked because no one model is statistically different from one another.

For each classification model, Figures S1-S3 show the ROC and PR curves with the highest means AUC-ROC and AUC-PR for each algorithm.


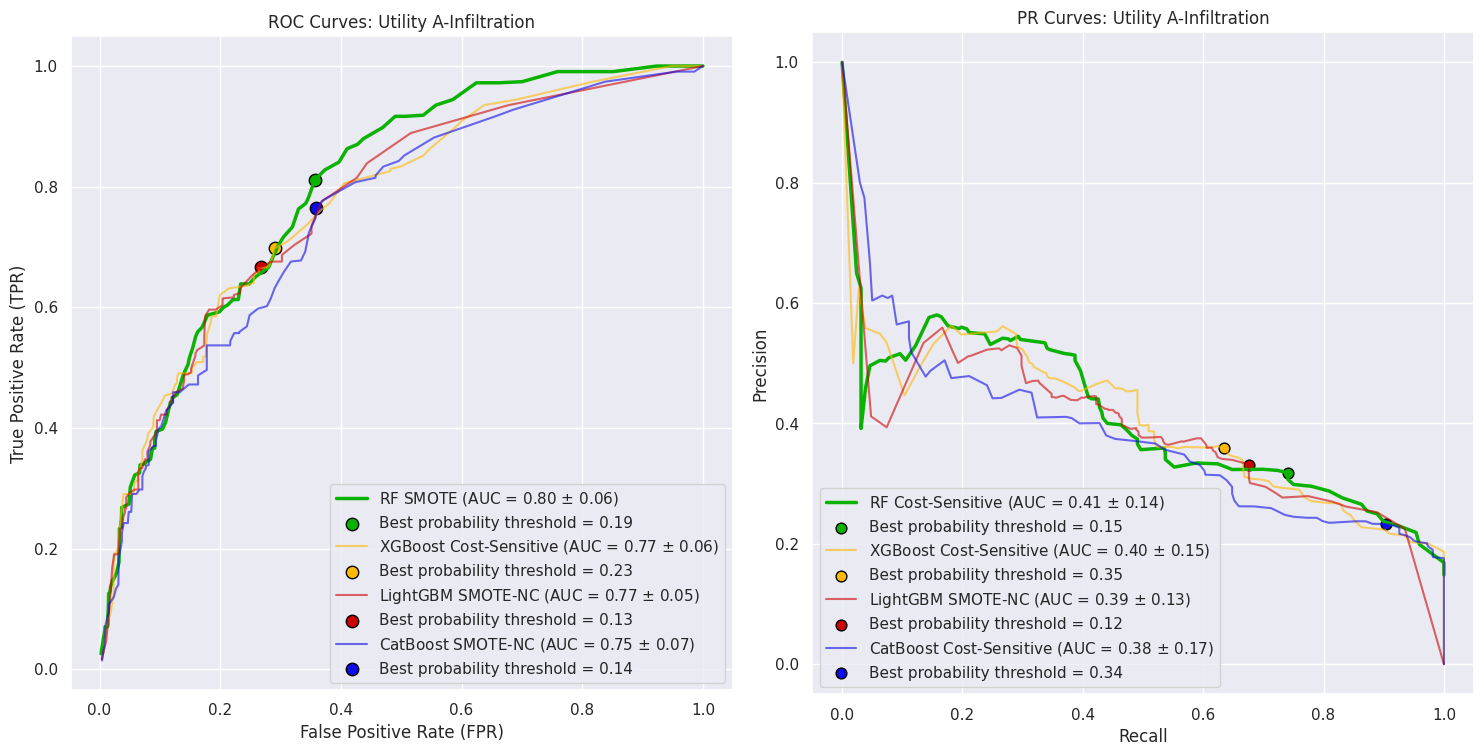


a)

b)

FIGURE S1. a) ROC and b) PR curves of Utility A infiltration classification models with the highest means AUC-ROC and AUC-PR for each algorithm.


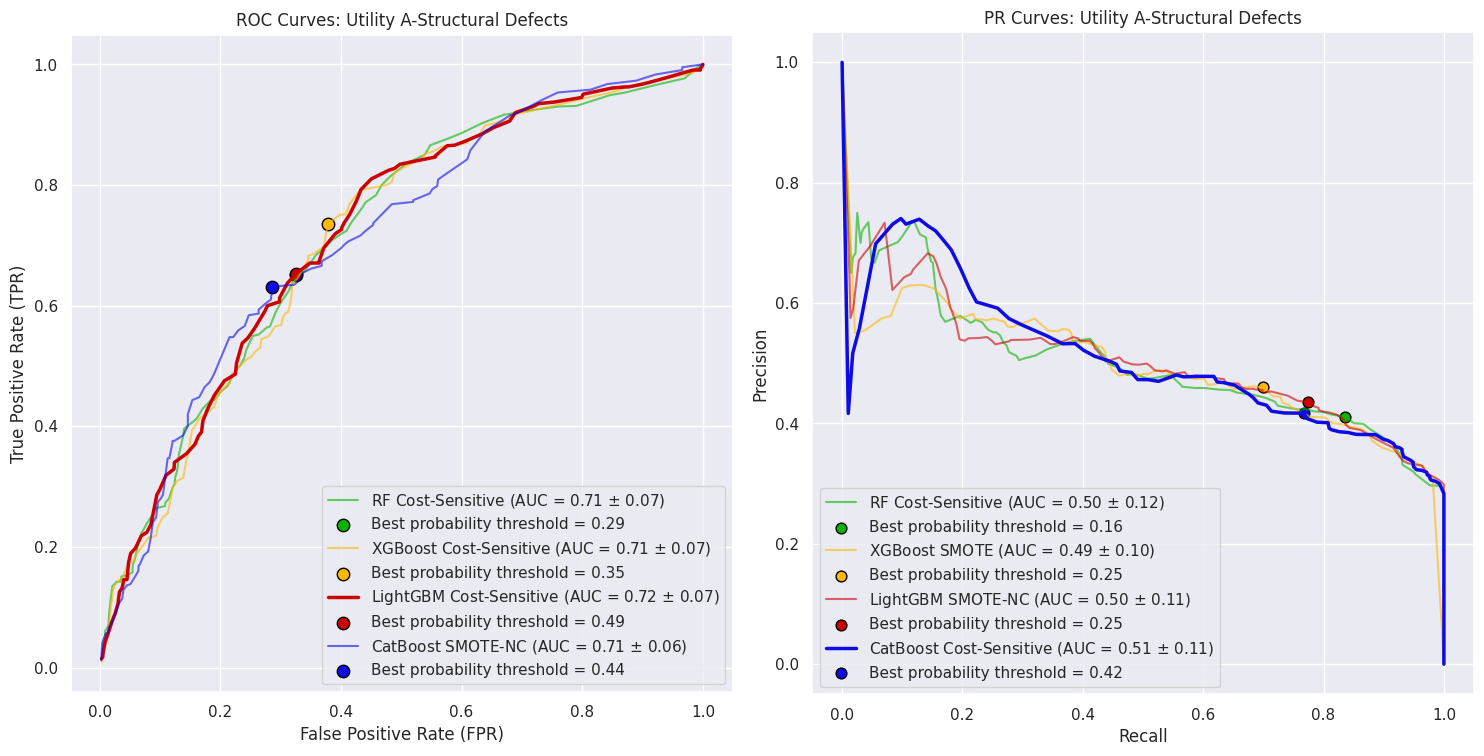


a)

b)

FIGURE S2. a) ROC and b) PR curves of Utility A structural defects classification models with the highest means AUC-ROC and AUC-PR for each algorithm.


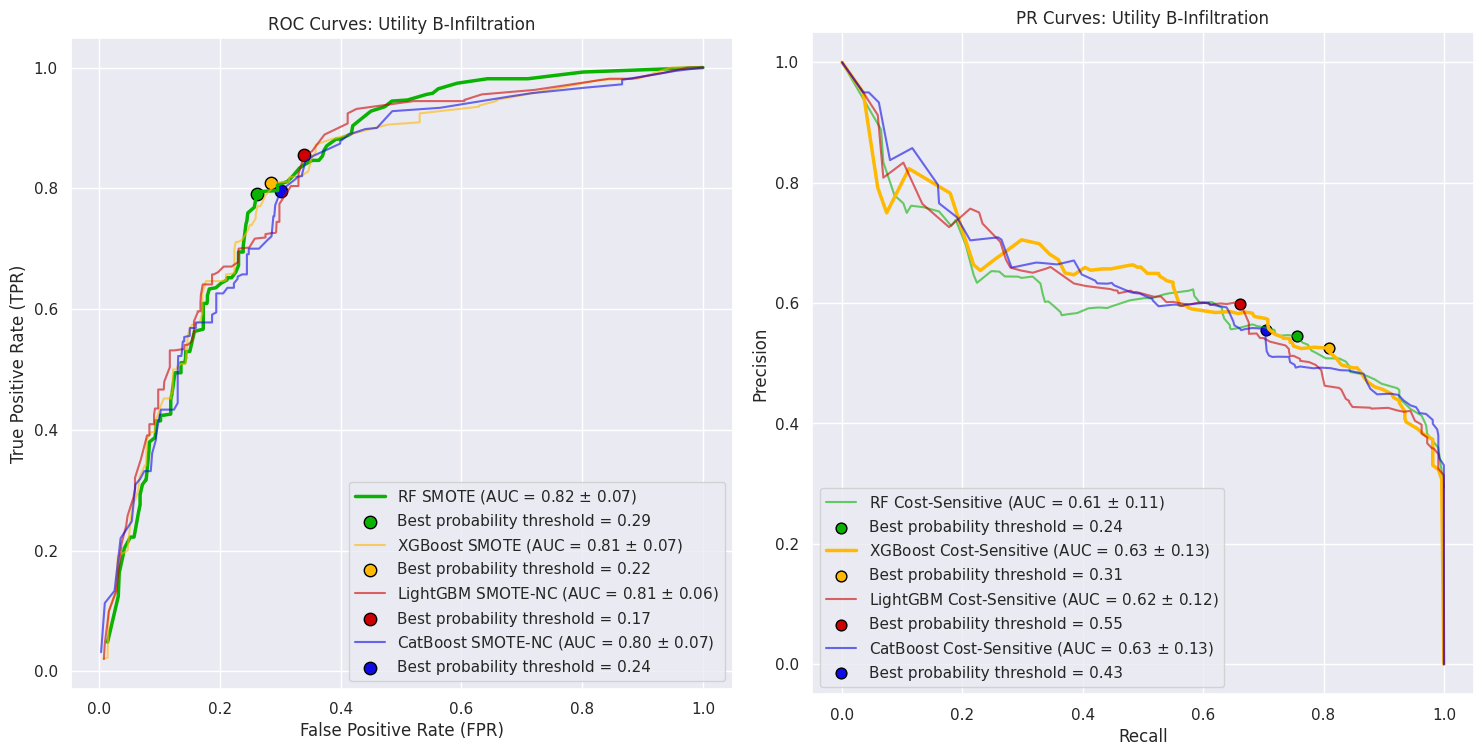


b)

a)

FIGURE S3. a) ROC and b) PR curves of Utility B infiltration classification models with the highest means AUC-ROC and AUC-PR for each algorithm.

## Shapley Additive exPlanations (SHAP) Analysis and Partial Dependence Plots

Figure S4 and Figure S5 show the SHAP summary plots for Utilities A and B, respectively, obtained from LightGBM-based models with the cost-sensitive learning method for addressing class imbalance. Each predictor variable is sorted by its importance from the top (the most importance) to the bottom (the least importance).


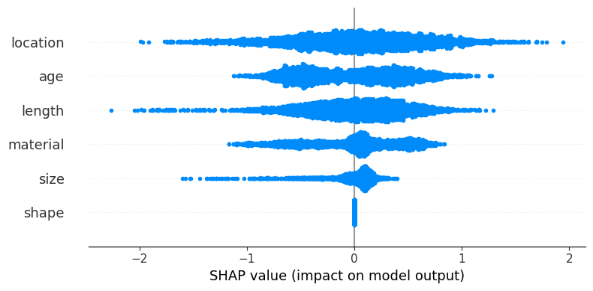

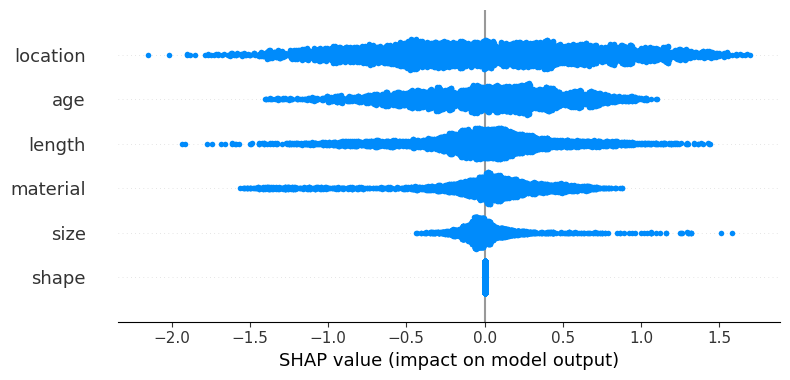


a)

b)

FIGURE S4. Model predictor variable importance for a) infiltration and b) structural defects classification models for Utility A.


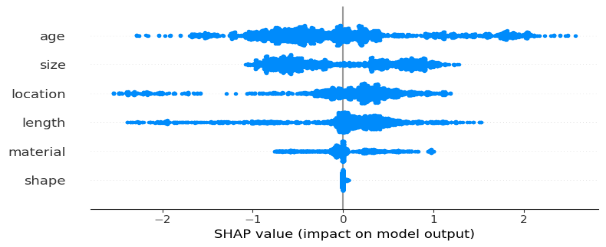

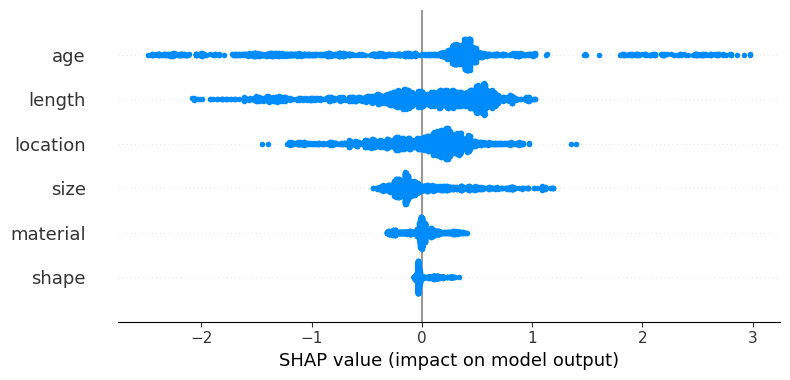


b)

a)

FIGURE S5. Model predictor variable importance for a) infiltration and b) structural defects classification models for Utility B.

The order of importance of the predictor variables for SHAP analyses of each model can be varied and are summarised in Table S8.

TABLE S8. Rank ordered model predictor variables from first to last place of each classification model

| Utility | Target variable | Algorithm | Imbalanced data managing technique | Rank ordered model predictor variables |
| --- | --- | --- | --- | --- |
| Utility A | Infiltration | RF | Cost-sensitive | location, length, age, material, size, shape |
|  |  |  | RUS | location, material, length, age, size, shape |
|  |  |  | SMOTE | location, material, age, size, length, shape |
|  |  | XGBoost | Cost-sensitive | location, length, age, material, size, shape |
|  |  |  | RUS | location, age, length, material, size, shape |
|  |  |  | SMOTE | location, material, length, age, size, shape |
|  |  | LightGBM | Cost-sensitive | location, age, length, material, size, shape |
|  |  |  | RUS | location, length, age, material, size, shape |
|  |  |  | SMOTE-NC | location, size, age, material, length, shape |
|  |  | CatBoost | Cost-sensitive | location, material, age, length, size, shape |
|  |  |  | RUS | material, location, length, age, size, shape |
|  |  |  | SMOTE-NC | location, material, size, age, length, shape |
|  | Structural defects | RF | Cost-sensitive | location, material, age, length, size, shape |
|  |  |  | RUS | location, material, age, length, size, shape |
|  |  |  | SMOTE | location, material, age, length, size, shape |
|  |  | XGBoost | Cost-sensitive | location, age, length, material, size, shape |
|  |  |  | RUS | location, age, length, material, size, shape |
|  |  |  | SMOTE | location, length, material, age, size, shape |
|  |  | LightGBM | Cost-sensitive | location, age, length, material, size, shape |
|  |  |  | RUS | location, age, length, material, size, shape |
|  |  |  | SMOTE-NC | location, age, length, material, size, shape |
|  |  | CatBoost | Cost-sensitive | material, age, length, location, size, shape |
|  |  |  | RUS | material, age, location, length, size, shape |
|  |  |  | SMOTE-NC | location, material, length, age, size, shape |
| Utility B | Infiltration | RF | Cost-sensitive | location, age, size, length, material, shape |
|  |  |  | RUS | size, age, location, length, material, shape |
|  |  |  | SMOTE | size, location, age, length, material, shape |
|  |  | XGBoost | Cost-sensitive | age, size, location, length, material, shape |
|  |  |  | RUS | age, size, length, location, material, shape |
|  |  |  | SMOTE | size, age, location, length, material, shape |
|  |  | LightGBM | Cost-sensitive | age, size, location, length, material, shape |
|  |  |  | RUS | age, size, length, location, material, shape |
|  |  |  | SMOTE-NC | age, size, location, length, material, shape |
|  |  | CatBoost | Cost-sensitive | size, age, length, location, material, shape |
|  |  |  | RUS | size, age, length, material, location, shape |
|  |  |  | SMOTE-NC | location, age, size, length, material, shape |
|  | Structural defects | RF | Cost-sensitive | location, age, length, size, shape, material |
|  |  |  | RUS | age, length, location, material, size, shape |
|  |  |  | SMOTE | location, age, length, shape, size, material |
|  |  | XGBoost | Cost-sensitive | age, length, location, size, material, shape |
|  |  |  | RUS | age, length, location, material, size, shape |
|  |  |  | SMOTE | age, length, location, shape, size, material |
|  |  | LightGBM | Cost-sensitive | age, length, location, size, material, shape |
|  |  |  | RUS | age, length, size, location, material, shape |
|  |  |  | SMOTE-NC | age, length, location, size, material, shape |
|  |  | CatBoost | Cost-sensitive | age, length, location, material, size, shape |
|  |  |  | RUS | length, age, material, shape, size, location |
|  |  |  | SMOTE-NC | length, age, location, size, material, shape |

Figures S6a-d and S7a-d are SHAP dependence plots of pipe age (a), length (b), material (c), and size (d) for structural defects for Utilities A and B, respectively.


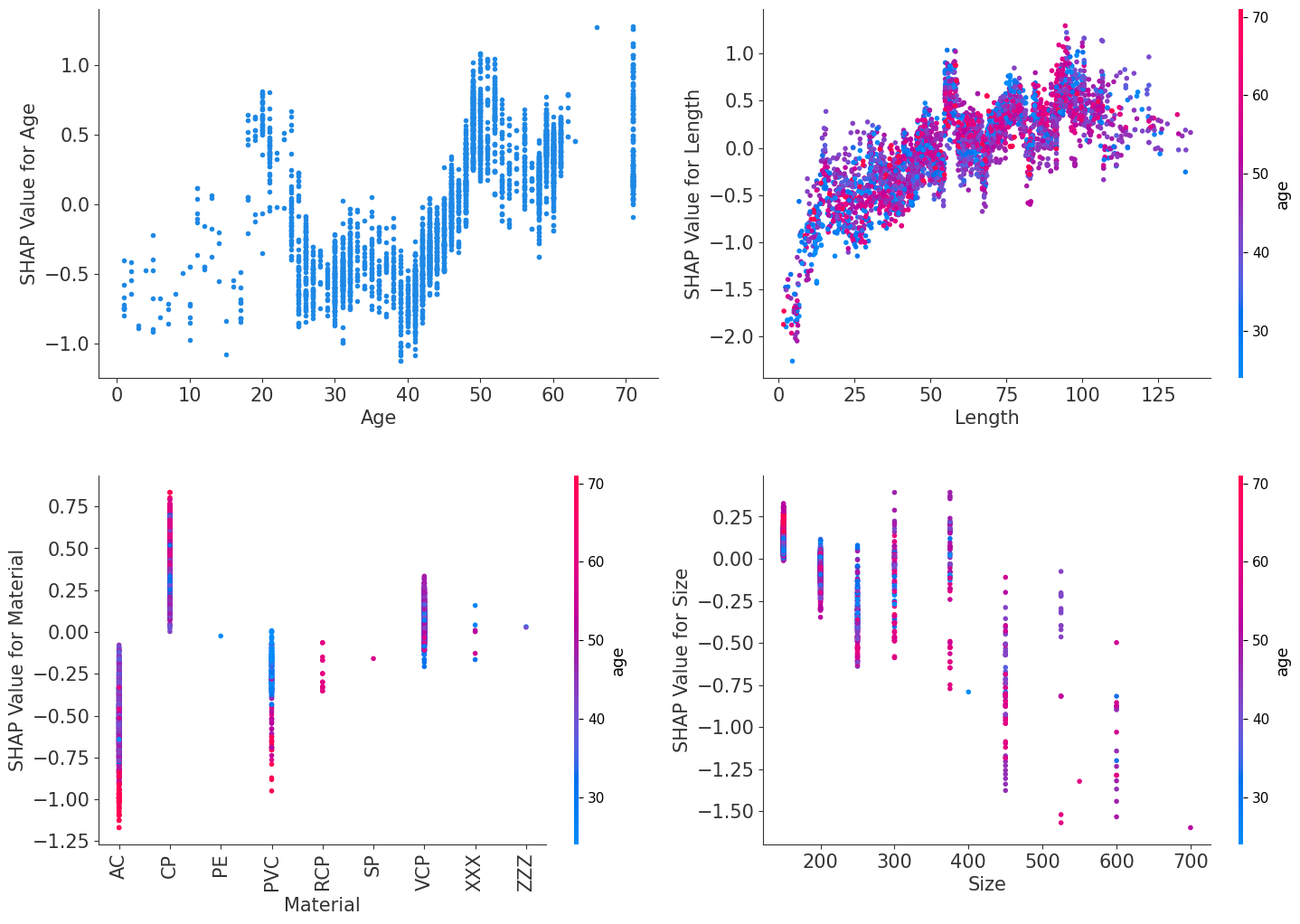


d)

c)

b)

a)

FIGURE S6. SHAP dependence plots for Utility A structural defects classification model for: a) pipe age; b) length; c) material; and d) size.


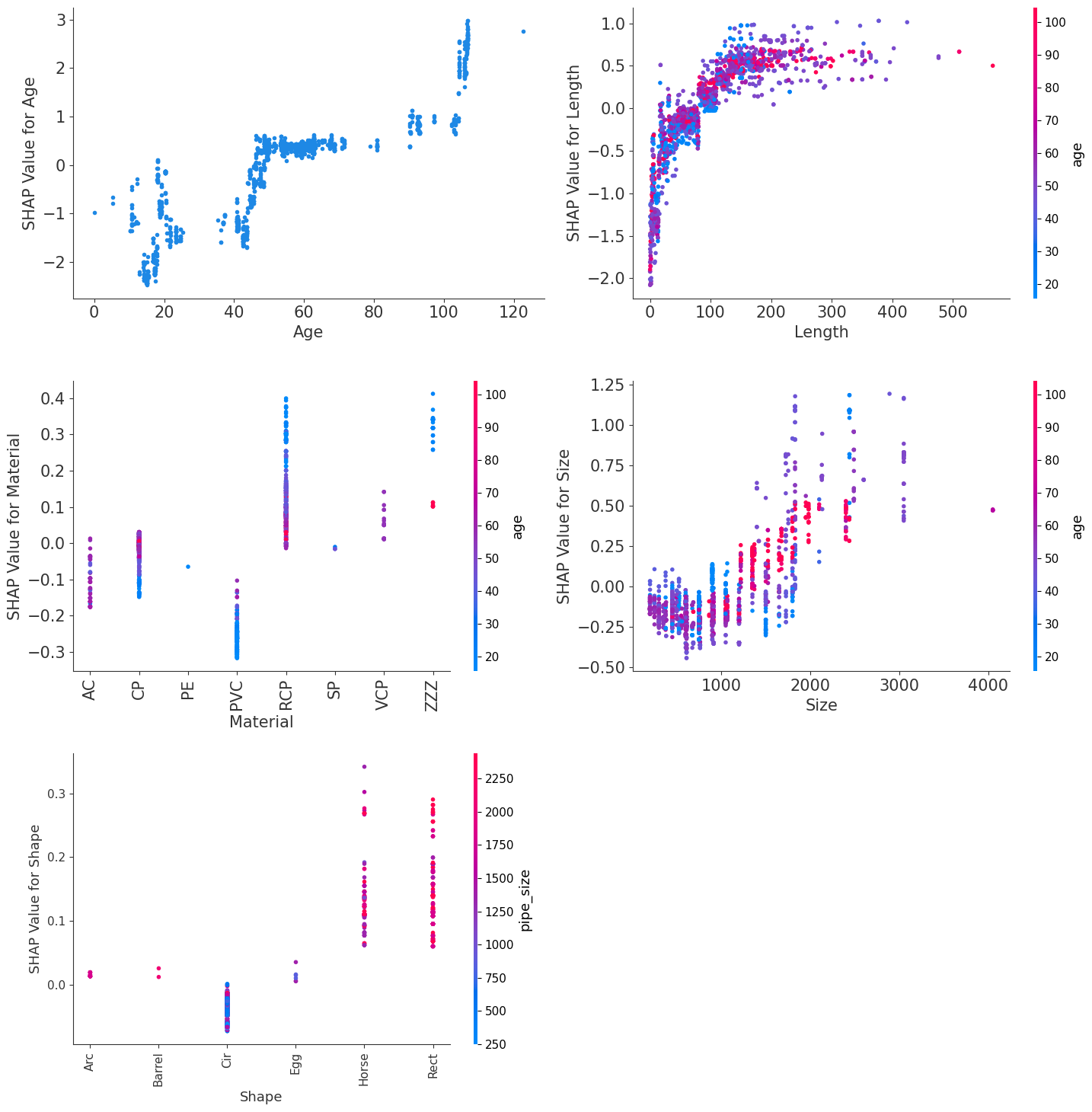


d)

c)

b)

a)

FIGURE S7. SHAP dependence plots for Utility B structural defects classification model for: a) pipe age; b) length; c) material; and d) size.

Figure S8 shows the likelihood that infiltration and structural defects are found in Utility B, denoted by numbers and colors, where the lighter colors represent the higher likelihood.


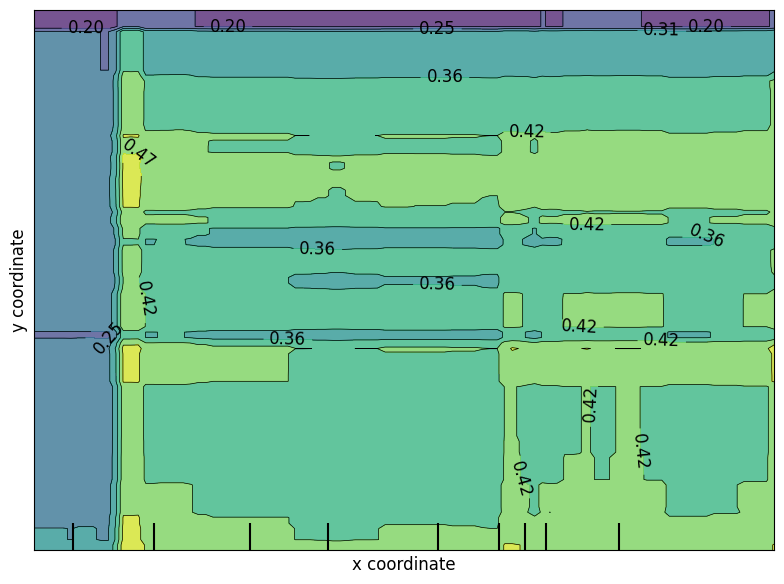

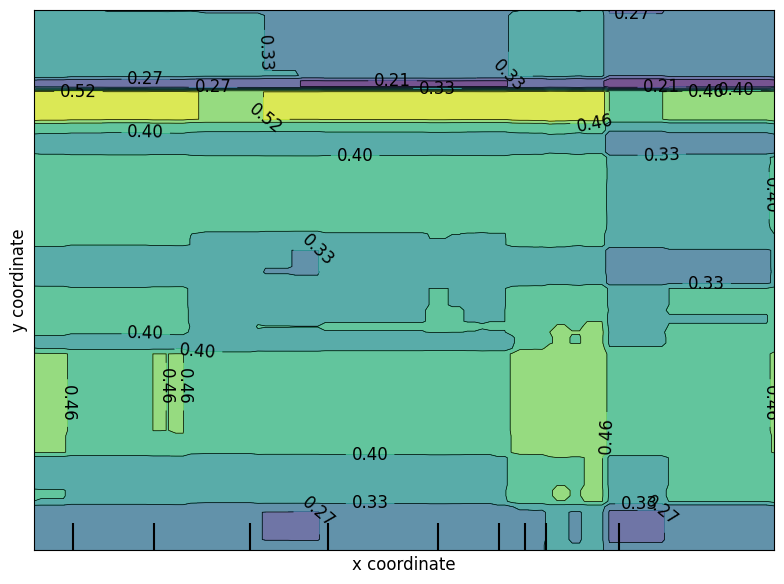


b)

a)

Figure S8. Partial dependence plot for x- and y- coordinates, likelihood of a) infiltration and b) structural defects for Utility B where lighter colors represent a larger likelihood while darker colors suggest a lower likelihood.
